# Recent Advances in Optical Sensing for the Detection of Microbial Contaminants

**DOI:** 10.3390/mi14091668

**Published:** 2023-08-26

**Authors:** Neslihan Idil, Sevgi Aslıyüce, Işık Perçin, Bo Mattiasson

**Affiliations:** 1 Department of Biology, Biotechnology Division, Hacettepe University, Ankara 06800, Turkey; nsurucu@hacettepe.edu.tr; 2 Department of Chemistry, Biochemistry Division, Hacettepe University, Ankara 06800, Turkey; sevgi@hacettepe.edu.tr; 3 Department of Biology, Molecular Biology Division, Hacettepe University, Ankara 06800, Turkey; ipercin@hacettepe.edu.tr; 4 Department of Biotechnology, Lund University, 22100 Lund, Sweden; 5 Indienz AB, Annebergs Gård, 26873 Billeberga, Sweden

**Keywords:** microbial contaminants, optical sensing, molecular imprinting, nanomaterials

## Abstract

Microbial contaminants are responsible for several infectious diseases, and they have been introduced as important potential food- and water-borne risk factors. They become a global burden due to their health and safety threats. In addition, their tendency to undergo mutations that result in antimicrobial resistance makes them difficult to treat. In this respect, rapid and reliable detection of microbial contaminants carries great significance, and this research area is explored as a rich subject within a dynamic state. Optical sensing serving as analytical devices enables simple usage, low-cost, rapid, and sensitive detection with the advantage of their miniaturization. From the point of view of microbial contaminants, on-site detection plays a crucial role, and portable, easy-applicable, and effective point-of-care (POC) devices offer high specificity and sensitivity. They serve as advanced on-site detection tools and are pioneers in next-generation sensing platforms. In this review, recent trends and advances in optical sensing to detect microbial contaminants were mainly discussed. The most innovative and popular optical sensing approaches were highlighted, and different optical sensing methodologies were explained by emphasizing their advantages and limitations. Consequently, the challenges and future perspectives were considered.

## 1. Introduction

Bacteria are commonly distributed and diverse microorganisms that can be found in fresh and salt water, sewage water, plants, animals [1], as well as human carriers not showing any signs of disease [2], seafood [3], and fly and insect larvae [4]. In the meantime, harmless bacterial strains, including *Salmonella*, *Escherichia coli*, *Staphylococcus*, etc., are important pathogenic microorganisms, and their spread needs to be prevented in hospitals and other healthcare settings [5,6]. On the other hand, the population of opportunistic bacterial strains increases with the weakened immune systems of individuals under the influence of some factors that result in increased susceptibility to infections [7]. After a long period of time, resistance to antimicrobial agents seems to be on the rise due to the accelerated number of patients hospitalized in intensive care units and the increasing number of compromised patients with immune deficiency, along with the unnecessary and inappropriate use of antimicrobial drugs [8]. Frequent usage of them, especially for both inpatients and outpatients, contributes to the development of resistance [9]. In this context, the data obtained from the U.S. Centers for Disease Control and Prevention shows that 2.8 million infections are reported in the U.S. each year [10], and 1.27 million deaths originated from antibiotic-resistant bacterial strains were reported around the world [11]. In the meantime, failure of the treatment leads to an increase in both treatment time and costs [12].

Food safety is one of the most significant and necessary public health problems worldwide [13]. Food-borne diseases emerging from consuming contaminated foods/water and food spoilage have been costly global troubles if necessary precautions are not taken [14]. Prevention of foodborne diseases relies on the careful processing of raw products; these diseases commonly arise from non-followed safety rules at food processing stages, which result in microbial contamination. Consumption of pathogenic microorganisms that contaminate food and/or intoxication with their toxic products are the origins of food-borne diseases, which are characterized by gastrointestinal symptoms [15]. Among the bacterial strains responsible for food-borne diseases that arise from milk, dairy, and meat products, *Staphylococcus aureus*, *Listeria monocytogenes*, and *Salmonella* sp. can be indicated as major examples [16]. From the point of rapid detection of these strains, it remains a challenge because of the diversity of bacteria and their presence in the complex media of the environment. It is necessary to detect these pathogenic microorganisms using a reliable, rapid, and cost-effective technology. Water-borne diseases are another global threat, leading to more than 2.2 million deaths per year, and their prevalence is increasing day by day [17]. In addition to deaths, morbidity is a significant result emerging from the consumption of contaminated water [18]. Therefore, there is a growing need to take control precautions for developing the security of drinking water, such as qualitative and quantitative microbial risk assessments [19]. Water-borne pathogenic microorganisms cause diarrhea, gastrointestinal problems, and systematic problems. To give examples, *Salmonella typhimurium*, *Vibrio cholerae*, *Legionella*, *E. coli* O157:H7, and *Campylobacter jejuni* are attributed to being major agents responsible for water-borne diseases [17]. 

The widely applied traditional microbiological detection methods are based on microbial cultivation techniques, biochemical tests, and serological tests that rely on antigen-antibody interactions, including enzyme-linked immunosorbent assay (ELISA), nucleic acid hybridization, and amplification methods. Some of these tests are manual, and some of them are semi-automatic or fully automatic. One of the most important points to be made is that they have limitations, such as being time-consuming. The incubation period changes according to the target bacterial strain and takes time (18–72 h) to determine the cell concentration. On the other hand, some microbial contaminants such as *V. cholerae* and *Helicobacter pylori* can be found in food, water, and the environment; they can be viable, but they cannot be easily cultivated [20]. The other limitations of the methods mentioned above can be listed as being open to contamination with other microorganisms, expensive, non-specific, and non-sensitive, and giving false positive or negative results. In addition, there is a necessity for trained personnel and pre-treatment stages [20,21].

Sensor technology looks forward to overcoming these drawbacks. Biosensors have been defined as analytical devices that convert biological responses into measurable signals. In this respect, firstly, the immobilization of ligands onto the sensor surface was performed to detect the target molecule. Then the sensing platform converts the response, which is produced by specific interactions carried out on the sensor surface, into physical signals. Lastly, a signal detector offers the advantage of monitoring the sensing. This technology plays a vital role in recognizing microbial contaminants with a rapid, sensitive, and selective assay. The limits of quantification (LOQ) and detection (LOD) help to explain the sensitivity of the sensing platform [20]. 

The use of optical sensing has recently gained great attention for chemical analysis [21], especially environmental monitoring [20], biomedicine [22], clinical [23], and industrial processes. This technology is frequently used to detect microbial contaminants in food [24], water [25], and the environment [26]. One of the most desirable features of optical sensing is obtaining stable measurements over a long period of time; therefore, they have been constructed for several purposes in the last quarter of a century due to their remarkable flexibility [27]. 

Optical sensing platforms offer alternative approaches, and newly developed sensing systems have focused on different types of optical sensors. Food-borne pathogens have been detected using optical based sensing in many studies. An aptamer based colorimetric biosensor was generated for the detection of *S. aureus* in a bacterial culture suspension using DNA as a recognition element, and the LOD was found to be 81 CFU/mL in 5.5 h [28]. In another study, a magnetic DNAzyme-copper-based fluorescent biosensor was produced for *E. coli* O157:H7 recognition in apple juice using protein as the recognition element, and the LOD was reported as 1.57 CFU/mL in 1.5 h [29]. Masdor et al. conducted a study aimed at detecting *Campylobacter jejuni* in chicken samples using antibody based SPR sensors [30]. Raghu and Kumar fabricated a Surface Plasmon Resonance (SPR) sensor to detect *L. monocytogenes* in milk samples using carbohydrate-lectin (Wheat germ agglutinin) interactions [31]. Waterborne pathogen detection using optical sensors is another interesting research area. Apart from these, cholera toxin, *Lactobacillus* sp., and *S. aureus* were detected by nanoparticle based colorimetric sensors [32]. In a study, *E. coli* was detected by a magnetic nanoparticle (MNP) based plasmonic sensor, and the LOD was found to be 3 × 10^3^ CFU/L [33]. In another study, *E. coli* was detected with iron oxide MNPs integrated with gold nanoparticles, and the LOD was reported as 8 × 10^3^ CFU/L in 70 min [34]. 

The related literature shows that label-free methods are more advantageous in comparison to labeled sensing methods, and label-free ones were preferred to obtain a quantifiable signal in the detection of microbial contaminants. From this point of view, labeling process is time-consuming and may have an effect on the physiological characteristics of microorganisms. On the contrary, label-free sensing strategies offer rapid and low-cost detection, simple preparation, and much more applicable assays on clinical and environmental platforms [35]. 

Electrochemical sensing platforms have also gained great attention for the detection of microbial contaminants. They offer relative simplicity, ease of design, low LOD, quick response, high sensitivity, and enhanced selectivity [36]. From the perspective of optical sensing, colorimetric sensing provides low-cost production, while plasmon sensing requires relatively expensive and complicated equipment. They are usually relatively simple in their construction. Besides they allow monitoring the target molecule with the help of visible light, which is easier to use than a power supply [37].

Many attempts were made to detect microbial contaminants using optical sensing such as colorimetric [38], fluorescence [39], interferometric [40], SPR [41], Raman spectroscopy [42], and SERS [43]. This area has taken on current interest and is attractive to be explored by scientists. The receptors, ligands, nanomaterials, molecularly imprinted polymers (MIPs), and hydrogels can be given as examples for the enhancement of the sensing platform’s effectiveness by providing high exactness, specificity, sensitivity, cost-effectiveness, low LOD, and rapid detection. In this review, the most promising strategies for optical sensing and recent advances are described. Besides polymeric materials integrated into the optical sensing platforms were discussed. In this respect, nanoparticle-based, MIP-based, and hydrogel-based optical sensing methodologies applied for the detection of microbial contaminants were explained. Representative, innovative, and popular examples from the literature were considered.

## 2. Optical Sensing

Optical sensors are based on the measurement of changes in the optical properties of a material. Binding of the target analyte with the recognition molecule causes a detectable optical change, and then this optical change is converted into an electronic signal [44]. Transducers in optical biosensors can be fluorescence, colorimetric, interferometric, or luminescent. Optical biosensors are promising technologies for detecting whole microbial cells. Optical biosensors mainly include (i) fluorescence-based sensors using secondary fluorescently labeled molecules that can bind bacteria specifically and (ii) label-free sensors such as SPR, which are able to detect refractive index change on the sensor surface according to bacteria amount [45]. 

Optical biosensors offer some advantages, such as high sensitivity, reliability, rapid measurement, direct measurement, and real-time measurement [46]. They are introduced as attractive alternatives for the analysis of many analyte types with low-cost and miniaturized sensing platforms. The response of the sensing system is related to the size of the analytes. Label-free sensing platforms were developed for small molecules. There are some limitations in this context, and immobilization methods were improved to overcome these problems.

### 2.1. Colorimetric Optical Sensors

Colorimetric optical sensors are developed as a result of distinctive color changes resulting from the reaction of the reagent and the analyte, which is selective to a specific analyte. In this context, the reagent has to be fixed on a suitable matrix [47]. This approach is promising for developing simple and low-cost microbial detection strategies and creating POC devices that provide a sensitive and selective response [38]. However, their weak-sensitivity property makes them poor tools. In a previous study, *E. coli* was detected with the naked eye using colorless 3,5,3′,5′ tetramethyl benzidine (TMB). Its color changes to blue from oxidized TMB in *E. coli* spiked medium [48]. Covalent binding, electrostatic interactions, and nano-films can be good candidates for stable docking states [49,50]. 

A simple and sensitive colorimetric aptasensor was developed to detect *Salmonella enterica serovar typhimurium*. ZnFe_2_O_4_-reduced graphene oxide nanostructures were prepared as effective peroxidase mimetics. ZnFe_2_O_4_-reduced graphene oxide nanostructures were conjugated with aptamers to obtain specific recognition elements. ZnFe_2_O_4_-reduced graphene oxide nanostructures can catalytically oxidize 3,3′,5,5′-tetramethylbenzidine (TMB) by H_2_O_2_ and produce a blue light that can be detected by a micro-reader at 652 nm. The detection range of *S. typhimurium* in buffer solution was 11 to 1.10 × 10^5^ CFU/mL, and the LOD was 11 CFU/mL [51]. 

A novel paper-based biosensor was developed for the detection of *S. aureus*. The mechanism originated from the proteolytic activity of *S. aureus*. *S. aureus* proteases showed activity on a specific peptide substrate that was placed between magnetic nanobeads and a gold surface on a piece of paper. The color change resulted from the dissociation of magnetic nanobeads and could be detected by the naked eye. Detection limits with visual observation were 7, 40, and 100 CFU/mL for *S. aureus* in broth culture and in inoculated food samples and environmental samples, respectively [52].

### 2.2. Fluorescence Optical Sensors

Fluorescence techniques using many organic fluorescence probes are designed to overcome the disadvantages of conventional microbial detection methods. Fluorescence-based small organic probes are easily designed and can provide real-time imaging and quantitative detection of bacteria cells in vitro and in vivo. Small organic probes can detect bacterial concentration due to fluorescence intensity, are highly selective, and sensitive, and can be applied to many applications. However, fluorescence probes are less biocompatible due to dye use and require cumbersome spectrophotometers. Furthermore, fluorophores have a short lifespan and may lose their photo-stability and recognition capability [53,54]. 

Elahi and his colleagues developed a fluorescent DNA sensor to detect Gram-negative *Shigella bacteria* that cause diarrhea. They used DNA probes and combined AuNPs with MNPs. The method detected low concentrations of bacteria (10^2^ CFU/mL) [55]. Pathak and his colleagues reported nitrogen-doped carbon-dot-based optical detection of pathogenic bacteria such as *E. coli*, *S. aureus*, *B. subtilis*, and *P. vulgaris*. The interaction of nitrogen-doped carbon dots with bacterial cells was pH-sensitive, and the best result was obtained at pH 2. Nitrogen-doped carbon dots (CDs) exhibited an intense photoluminescent emission at λex/λem = 340/435 nm, and their calculated quantum yield was 27.2% [56]. 

*Acinetobacter baumannii* was detected in a urine sample by a graphene oxide based fluorescent aptasensor method developed by Bahari and his colleagues. *A. baumannii* causes pneumonia and urinary and blood infections. This ratiometric fluorescent aptasensor method used o-CD/nitrogen-doped-CD as donors and graphene oxide as acceptors in the FRET process. *A. baumannii* detection was applied in the concentration range of 2.0 × 10^3^–4.5 × 10^7^ CFU/mL, and a low detection limit (3.0 × 10^2^ CFU/mL) was obtained [57]. 

### 2.3. Interferometric Optical Sensor

Interferometric sensors are based on an optical method for measuring refractive index changes. Biomolecular interactions such as antigen-antibody, enzyme-substrate, or DNA hybridization and chemical reactions cause a change in refractive index [38]. An interferometric sensor can be applied to detect bacterial cells by providing the right choice of sensing film. The basic principle of an interferometer is that it uses two equivalent light paths. One light gives the refractive index change caused by bioconjugate interaction, and the other light acts as a reference that equalizes nonspecific interactions. Interferometers are highly sensitive and can detect refractive index changes of 10^−7^. These refractive index change values correspond to 100–1000 s of whole bacterial cells, viruses, pg/mL concentrations of proteins, and ppb concentrations of small molecules [58]. 

A sensor platform was developed that relies on label-free long-period fiber gratings for the recognition of *S. aureus*. In this respect, polyelectrolyte modification was performed to create specificity for the target microorganism. The sensing system was evaluated according to the time-resolved resonance wavelength shift, and the LOD was found to be 224 CFU/mL within 30 min [59]. 

Janik et al. developed a label-free and microcavity in-line MZI sensor for the detection of *E. coli* O157:H7 with the use of peptide aptamers as bioreceptors. (3-Aminopropyl) triethoxysilane was preferred for the functionalization of the sensor surface, and then, aminosilane and peptide aptamers enabled detection of heat-treated *E. coli*. As a result, LOD was found to be 10 CFU/mL [60]. 

### 2.4. Plasmonic Optical Sensors

Plasmon resonance-based biosensing and chemical sensing platforms utilize the principle of forming plasmons with the interaction of light with electrons to create an electromagnetic wave on the surface. Surface plasmon resonance and localized surface plasmon resonance (LSPR) can be used in harsh conditions and provide rapid and sensitive, label-free detection of many types of bacteria. However, the large size of the SPR design and the special algorithmic needs of the smartphone-based approach are some disadvantages of plasmonic techniques [61,62].

SPR sensing systems include a polarized excitation light source, a prism, and a gold-film-coated sensor. The principle of SPR is based on the conversion of the refractive index change triggered by molecular binding into resonant spectral shifts. Polarized light passes through a prism in a thin metal-film-covered sensor chip; the light is reflected by the metal film, which acts like a mirror. The reflected light leads to a marked decline at a specific angle. In this context, upon changing the angle and monitoring the reflected light intensity, it is observed that the intensity of the reflected light passes through a minimum range. At the incidence angle, the reflected light stimulates surface resonance and induces plasmon resonance; therefore, the intensity of the reflected light decreases [63,64]. 

Bioreceptors such as antibodies, enzymes, nucleic acids, cells, bacteriophages, and biomimetic tools [65] and different transducers, which include electrochemical, optical, and mass-based detection, are widely applied for the recognition of microorganisms [66]. Nano-based optical sensing is another approach with desirable features such as rapid, easily prepared, and inexpensive detection. Nanomaterials have been verified to be effectively used in the functionalization of recognition surfaces to enhance the sensitivity of sensing platforms [67]. 

Antibodies can be used as recognition elements and combined with SPR sensing. To give an example, *E. coli* O157:H7 was detected with an antibody-based SPR system in the milk, apple juice, and ground beef extract samples [68]. Besides it was reported that immunosensor systems were preferred to enzyme labels for the detection of microbial contaminants in food. In this regard, *L. monocytogenes*, *E. coli*, and *C. jejuni* strains were detected using horseradish peroxidase enzyme integrated into sandwich immunoassays, including carbon particles [69]. In related literature, there are many publications indicating the widespread usage of optical sensing systems. Representative examples of the plasmon-based optical sensing platforms applied for the detection of microbial contaminants are given in Table 1. 

The localized surface plasmon resonance (LSPR) responds according to the diffraction index change of the medium. In this context, sensing relies on the sum of the absorption and reflection of light in metallic nanoparticles (i.e., silver and gold) showing a close field at resonance wavelength. The responsible responses can be monitored by observing the shift of the maximum peak wavelength using UV-Vis spectroscopy [70]. Gold nanoparticles (AuNPs) are one of the most preferred materials to apply for LSPR-based sensing due to their chemical stability and resistance to oxidation. Besides they provide an opportunity to obtain high sensitivity by sensing refractive index changes [71]. LSPR has been widely used in the fields of medicine, food and water safety, and environmental analysis due to its advantages of low-cost preparation, label-free, real-time, and rapid detection. Nano-structured molecules have great potential to enhance miniaturization, which enables the creation of POC devices [72].

**Table 1:** Representative examples of the plasmon-based optical sensing platforms applied for the detection of microbial contaminants.

Sensing Platform	Microbial Contaminant	Ligand	Sample	Concentration Range	LOD	Ref.
SPR	*Brucella melitensis*	B70 aptamer	Milk	10^2^–10^6^ CFU/mL	27 ± 11 cells	[73]
SPR	*L. monocytogenes*	Wheat germ agglutinin	Milk	1–8 log CFU/100 μL	3.25 log CFU/100 μL	[31]
SPR	*E. coli*	Polyclonal antibodies	Milk	10^5^–10^8^ CFU/mL	6.3 × 10^4^ cells	[74]
SPR	*E. coli* O157:H7 *Salmonella enteritidis*, *L. monocytogenes*	Polyclonal antibodies	Chicken	10^6^ CFU/mL	14 CFU/25 mL, 6 CFU/25 mL, 28 CFU/25 mL	[75]
SPR	Endotoxin	-	LAL test reagent	50–0.0005 EU/mL	<0.0005 EU/mL	[76]
SPR	*E. coli*	Antibodies	Aqueous solution	3.8 × 10^6^ to 9.76 × 10^8^ CFU/mL	1.1 × 10^6^ CFU/mL	[77]
LSPR	*S. aureus*	Aptamer	Milk	10^3^–10^8^ CFU/mL	10^3^ CFU/mL	[78]
LSPR	*E. coli* O157:H7	Antibodies	Fresh lettuce	10^1^–10^6^ cell/mL	10 cell/mL	[79]
LSPR	*E. coli* O157:H7	Antibodies	Aqueous solution	10^1^–10^5^ CFU/mL	10 CFU/mL	[80]
LSPR	*E. coli* O157:H7	Antibodies	Aqueous solution	10^1^–10^5^ CFU/mL	10 CFU/mL	[81]

SPR: surface plasmon resonance; LSPR: localized surface plasmon resonance.

### 2.5. Raman Spectroscopy

When a high-energy light beam is sent onto a substance, it passes through this permeable medium, and a small part of the incident light is scattered in different directions. It was discovered that the wavelength of a small amount of light scattered by some molecules is different from the wavelength of the incident ray. These wavelength shifts are related to the chemical structure of the molecules causing the scattering. In Raman scattering, results were obtained from inelastic light scattering on vibrating molecular bonds. Raman spectroscopy is defined as a powerful molecular fingerprinting technique that analyzes with the help of a laser beam, the interactions of molecules of matter. Although this technique has been widely used for the characterization of different materials, it has been rapidly found in many biological applications to provide optical detection of microorganisms [42,82]. 

In a previous study, surface imprinting was applied with a glass-stamping approach to covalently immobilize *E. coli* onto the poly(styrene-co-DVB). Confocal Raman Microscopy was used for monitoring bacterial recognition regions. Imprinting efficiency and selectivity of the proposed sensing platform depend on both Confocal Raman Microscopy and Partial Least Squares Discriminant Analysis (PLS-DA). The Raman spectra of *E. coli* and *Bacillus cereus* were obtained using *E. coli*-imprinted poly(styrene-co-DVB), and then a PLS-DA model was formed to discriminate different microorganisms. The verification of the model showed an accurate classification of 95% of Raman spectra, and therefore, selectivity experiments were successfully performed. It was reported that discrimination of *E. coli*, *B. cereus*, and *Lactococcus lactis* using *E. coli*-imprinted poly(styrene-co-DVB) was troublesome since restricted depth resolution of the confocal Raman microscope leads to the emergence of interfering signals originating from polymeric structures. It is considered that this problem can be handled with the application of type-enhanced Raman spectroscopy [83].

Only one in a million photons sent to the system undergoes Raman scattering, and this condition is not sufficient for the detection of target molecules at low concentrations. To overcome this challenge, many attempts were made to improve the system on the surface, such as the use of enhanced Raman spectroscopy (SERS). Since SERS allows the detection of target molecules at low concentrations, it is a much more precise technique than classical Raman. SERS is the form of Raman signals enhanced with SERS substrates [84]. It is a type of Raman spectroscopy based on the interaction between the substance to be analyzed and nano-scale gold or silver, copper surfaces, and/or colloids [85]. 

SERS is a spectroscopy technique that provides sensitivity to the single molecule level and allows rapid identification of target analytes such as disease markers, chemicals, and microorganisms [86]. Due to its high resolution, high sensitivity, and rapid data acquisition, SERS has become a hot research field for bacteria detection in water and food. The SERS technique allows the design of lab-based and portable instruments and can be applied to any surface on account of the involvement of scattered light. SERS have some disadvantages, such as unstable laser wavelength and intensity and/or interference caused by fluorescence, turbidity, and other molecules in the environment. Label-free and label-based SERS sensor strategies are used for bacteria detection [87]. Label-free methods allow direct detection of the Raman signal of bacterial cells and/or bacterial metabolites utilizing vibrational spectroscopy methods based on molecular fingerprints. The label-free method is simple and easy to apply; however, the weakness of the original Raman signal of the bacterial cell makes it a challenge to get highly reproducible spectral data. Label-based bacteria detection methods use SERS tags, which are able to bind bacteria through specific recognition sites and produce ultrasensitive and distinct signals. Thus, bacterial cells can be detected indirectly by determining the Raman signal of the reporters. When compared to the label-free method, the label-based SERS method is more sensitive and reproducible [88]. 

## 3. Material Used for Optical Sensing Platforms

### 3.1. Nanomaterial-Based Optical Sensing

Biosensors are divided into biocatalytic and bioaffinity-based platforms based on their recognition mechanisms. In biocatalytic systems, after the receptor binds to the analyte, it catalyzes the reaction that leads to its degradation. The bioaffinity system, the receptor, binds to the analyte and triggers the detection and formation of a signal [89]. NPs are materials that can be integrated into these sensing platforms. In recent years, the combined use of optical sensors to improve the rapid and sensitive detection of pathogens has gained momentum with the rapid developments in the field of nanotechnology. Nanomaterials have been promising tools for sensitive detection with much lower detection limits due to their large surface area. The large surface area provides a large surface for the binding of many biomolecules, such as enzymes, antibodies, or DNA sequences required for biological recognition. Besides they can be easily modified to achieve the desired properties, which makes them advantageous materials. 

Noble metals such as gold, silver, platinum, and palladium are highly resistant to oxidation and erosion caused by environmental factors [90]. Functional nanomaterials can be obtained by integrating these metals or their alloys into NPs [91]. Thus, they can remain stable in complex liquids in which microbial contaminants will be analyzed. These materials can exhibit different optical, catalytic, and magnetic properties depending on their different sizes and shapes. Thanks to their catalytic properties, noble metal NPs can show enzyme-like activities depending on reaction conditions, such as peroxidase in an acidic medium and catalase in a basic medium [92].

Noble metal NPs are used in detection platforms such as electrochemical, optical, colorimetric, chemiluminescence, and fluorescence-based sensors [93]. In colorimetric sensor applications, the color change during the detection of the target molecule can be seen even with the naked eye without using a different device with the help of noble metal NPs. Noble metal NPs also cause signal amplification and higher sensitivity in plasmonic sensor systems. When the surface of the SPR sensor chip is replaced with the noble metal NPs, light interacting with the noble metal NPs has dimensions much smaller than the incident wavelength. Thus, it leads to a localized SPR plasmon that is released around the nanoparticles [94].

Within the framework of this section, representative examples are given regarding optical sensor systems based on various nanomaterials such as AuNPs, magnetic nanoparticles (MNPs), quantum dots (QDs), CDs, and nanofibers for the recognition of microbial contaminants [95,96,97]. All these nanomaterials can be used alone, or they could be integrated into optical sensor platforms. Recent publications providing examples of nanomaterial-based optical sensing for the detection of microbial contaminants are shown in Table 2.

**Table 2:** Recent publications providing examples of nanomaterial-based optical sensing for the detection of microbial contaminants.

Composite Nanomaterial	Sensing Platform	Microbial Contaminant	Concentration Range	LOD	Ref.
Au NPs/Silica NPs	LSPR	*Lactobacillus acidophilus*, *S. typhimurium*, *Pseudomonas aeruginosa*	10^4^–10^12^ CFU/L	10^4^ CFU/L	[98]
MnO_2_ Nanoflowers/QDs	Fluorescent immunoassay	*E. coli*, *S. typhimurium*.	1.5 × 10^1^–1.5 × 10^6^ CFU/mL, 4 × 10^1^–4 × 10^6^ CFU/mL	15 CFU/mL, 4 CFU/mL	[99]
CDs/MNPs	Fluorescent detection	*E. coli*, *S. aureus*	10^2^–10^3^ CFU/mL	3.5 × 10^2^ CFU/mL 3 × 10^2^ CFU/mL	[100]
GO/QDs	Photoluminescent Lateral-Flow Immunoassay	*E. coli*	10 CFU/mL (standard buffer) 100 CFU/mL (bottled water and milk)	2–10^5^ CFU/mL	[101]
Ag/ZnO/rGO	SERS	*E. coli*	5 × 10^4^–5 × 10^8^ CFU/mL	10^4^ CFU/mL	[102]
Ag NPs/rGO	SERS	*E. coli*	1 × 10^5^–2 × 10^8^ CFU/mL	1 × 10^5^ CFU/mL	[103]
Au@Ag NP	SERS	*S. typhimurium*	10^1^–10^5^ cells/mL	15 CFU/mL	[104]
CDs-microspheres	Fluorescent immunoassay	*E. coli* O157:H7	2.4 × 10^2^–2.4 × 10^7^ CFU/mL	2.4 × 10^7^ CFU/mL	[105]
NBs/GNRs	SERS aptasensor	*E. coli* O157:H7	10–10,000 CFU/mL	3 CFU/mL	[106]
Dual-functional metal complex- AuNPs	SERS aptasensor	*Shigella sonnei*	10–10^6^ CFU/mL	10 CFU/mL	[107]
GQDs/GO	FRET immunosensor	*Campylobacter jejuni*	10–10^6^ cell/mL	10 cell/mL	[108]
CQDs-MNPs	Fluorescent aptasensor	*E. coli* O157:H7	500–10^6^ CFU/mL	487 CFU/mL	[109]
Rhodamine B modified silica NP	Fluorescent assay	*E. coli*	10–10^5^ CFU/mL	8 CFU/mL	[110]

LSPR: localized surface plasmon resonance, NPs: nanoparticles, QDs: quantum dots, CDs: carbon dots, MNPs: magnetic nanoparticles, GO: graphene oxide, rGO: reduced graphene oxide, SERS: surface enhanced Raman scattering, NBs: gold nanobones, GNRs: gold nanorods, FRET: fluorescence resonance energy transfer, CQDs: Carbon quantum dots.

#### 3.1.1. Gold Nanoparticles (AuNPs)

AuNPs can emit bright resonance light scattering at various wavelengths due to many properties, such as their size and degree of aggregation. The optical properties of AuNPs have emerged from their surface plasmon resonance properties. These particles can both absorb and scatter visible light. Along with the absorption of light, the light energy causes the free electrons in the gold nanoparticles to oscillate. As the particle gets larger, a greater proportion of the outgoing light is scattered compared to what is absorbed. As a result, solutions of gold nanoparticles of different sizes and shapes appear in different colors. AuNPs are frequently used as colorimetric sensors due to their optical properties. Awwal et al. prepared fluorescently labeled AuNP functionalized with an anti-*E. coli* O157:H7 antibody for the detection of *E. coli* O157:H7. They conjugated the antibody with Tetramethyl Rhodamine Isothiocyanate and then labeled it with the aminated gold nanoparticle. According to the results obtained from fluorimeter measurements in the emission range of 555–650 nm, the detection was performed in 5 min. The detection range of the immune-fluorescent biosensor was reported as 10^3^–10^5^ CFU/mL [111]. 

**Figure 1:**
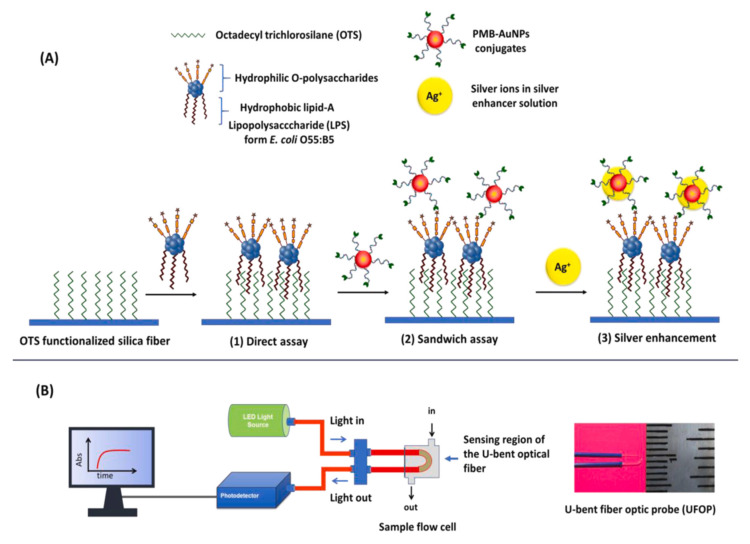
Schematic representation of (**A**) the functionalization steps for LPS detection and (**B**) the optical setup applied for LPS detection. Reproduced with permission from [112].

Endotoxins are in the class of lipopolysaccharides and are present on the surface of the cell wall of Gram-negative bacteria. Endotoxins can be found in the blood circulation system in severe bacterial infections. Thus, early diagnosis of septic shock is important, and early detection of endotoxins in medical devices and/or in the blood can save lives. Manoharan et al. used the plasmonic sandwich test with antibiotic conjugated AuNP labeling to improve endotoxin detection with fiber optic plasmonic sensors. The endotoxin is hydrophobically attached to the fiber optic probe. First, they prepared a U-bent fiber optic probe (UFOB) with octadecyltrichlorosilanes (OTS) function for real-time endotoxin detection. Then, they labeled the attached Endotoxin with Polymixin B-linked AuNP and measured the plasmonic absorption response by the sandwich assay. In the last stage, they enhanced the plasmonic sensor with silver to increase the sensitivity (Figure 1A). The optical setup of the UPOF is indicated in Figure 1B. Consequently, they reported that the sensitivity of the sensor was increased 36 times [112].

Another antibiotic assisted AuNPs research project for the detection of bacteria was performed by Song and coworkers. AuNPs were synthesized by the sodium citrate reduction method and then functionalized by treating them with 11-mercaptodecanoic acid. Afterwards, they activated AuNPs using N-hydroxy succinimide and 1-(3-dimethylaminopropyl)-3-ethylcarbodiimide hydrochloride. Finally, they performed the colorimetric detection of *Pseudomonas fluorescens* by binding amikacin to these AuNPs. According to the obtained data, LOD was found to be 10^4^ CFU/mL and 2.9 log CFU/mL with the naked eye and spectral analysis, respectively [113]. 

Verdoot et al. prepared an immunosensor for the detection of *S. aureus* and *Lactobacillus* sp. by immobilizing a monoclonal anti-Gram-positive bacterial antibody to the surface of AuNPs. Target bacterial strains were detected by the change in color and the UV-vis spectrophotometer. They reported the LOD for *Lactobacillus* sp. and *S. aureus* as 105 CFU/mL and 120 CFU/mL, respectively [114].

Sun et al. prepared a lateral flow strip for simultaneous detection of Gram-positive and Gram-negative bacteria with AuNPs. In the first step, they attached the primary recognition molecule, ampicillin antigens, to AuNPs. In the second step, they bond vancomycin and aptamer, which are secondary recognition molecules. Here, vancomycin recognizes gram-positive bacteria, and aptamer is specific for Gram-negative bacteria. They reported the detection limit of this lateral flow strip as 4 CFU/mL [115].

#### 3.1.2. Magnetic Nanoparticles (MNPs)

Sensing platforms are based on signal generation depending on the interaction between the analyte in the sample solution and the sensing surface. MNPs enable the collection of the analyte from the sample and provide mass transport to the sensing surface under magnetic conditions. In this context, the application of magnetic particles facilitates the pre-concentration of the sample, including the target analyte; therefore, a sensing response will be obtained more rapidly along with increased sensitivity. Another advantage is that MNPs provide the opportunity for temporal separation of the sample. Besides they can be easily coated and functionalized to construct sensing systems. Apart from these, the stability of the magnetic materials can be increased by preventing oxidation. Gold is one of the most common coating agents for MNPs used in optical sensing due to its optical properties [116].

**Figure 2:**
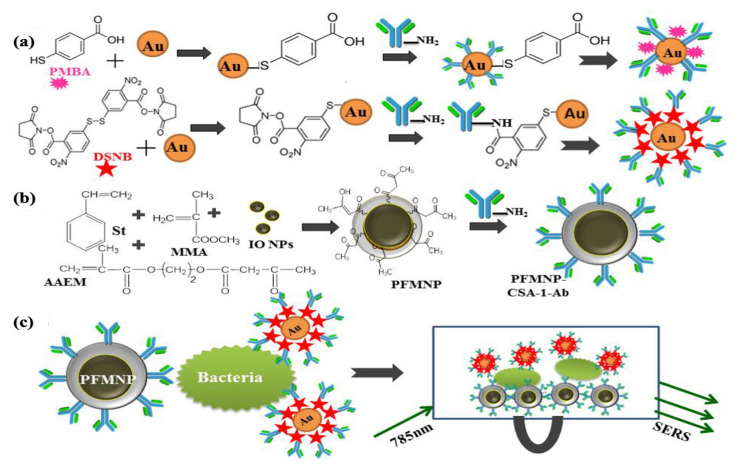
(**a**) Modification of synthesized GNPs with MBA and DSNB (**b**) Synthesis of FPMNPs and bioconjugation with CSA-1-Ab (**c**) Magnetically assisted capture of *S. typhimurium* and their detection using a SERS signal probe. Reproduced with permission from [43].

Chattopadhyay et al. prepared a SERS-based immunosensor functionalized with polymeric magnetic nanoparticles (PFMNPs) to detect *S. typhimurium*. AuNPs were preferred for use as signal probes. The signal properties of these two molecules were investigated by modifying AuNPs separately with 4-mercapto benzoic acid (MBA) and 5,5′-dithiobis succinimidyl-2-nitrobenzoate (DSNB), which are known as Raman reporter molecules (Figure 2a). CSA-1-Ab, a specific antibody against *Salmonella* antigen, was used to create specific recognition. CSA-1-Ab was bound to the PFMNP surface by the amino-ketoenol chemistry method. Styrene (St), methyl methacrylate (MMA), acetoacetoxy ethyl methacrylate (AAEM), and iron oxide nanoparticles were used for the synthesis of PFMNPs (Figure 2b). In this report, LOD values were found to be 100 cells/mL and 10 cells/mL for the MBA-based sensor and the DSNB-based sensor, respectively. The impressive property of the resultant sensing system is that the magnetic character of the polymeric structure enables the pre-enrichment step, and it could be an effective candidate to produce reliable SERS signals (Figure 2c) [43]. 

Wang et al. developed a smartphone-based fluorescence imaging system to detect *S. typhimurium*. First, the magnetic nanoparticles (MNPs) were modified with monoclonal antibodies and *S. typhimurium* attached to the nanoparticles. The bacterial and magnetic nanoparticle complexes were then labeled with fluorescent and polyclonal antibody-modified microspheres (FMSs). A calibration curve was drawn with measurements at concentrations in the range of 1.4 × 10^1^–1.4 × 10^6^ CFU/mL. The selectivity of the sensor was verified by the examination of the sensing responses of four different bacterial strains other than the target microorganism. In conclusion, LOD was reported as 8 CFU/mL for *S. typhimurium* [117].

A SERS-based methodology was applied for the sensitive detection of *S. typhimurium*, which was reported by Yang and coworkers. This developed method is based on a three-dimensional DNA walker model using gold modified magnetic nanoparticles (AuMNPs). In this context, the PolyA-DNA sequence was bound to AuMNPs and paired with the complementary aptamer (cApt) sequence. Thus, the DNA walker reaction is triggered by a nicking endonuclease. Next, the SERS tag specifically binds to DNA fragments on the surface of AuMNPs. As a result, the LOD was found to be 4 CFU/mL for the proposed SERS-based detection method for *S. typhimurium* detection [118].

#### 3.1.3. Quantum Dots (QDs)

QDs are nano-sized semiconductor crystals. Depending on their size, they have an adsorption spectrum at all visible wavelengths and even in the ultraviolet. These unique optical properties of QDs make them suitable tools for their use as sensitive and selective fluorescent probes and labels for many bioassays. Cui et al. designed a fiber optic probe and QDs-based immunofluorescence sensor to detect *S. aureus*. First, the rabbit polyclonal anti-*S. aureus* antibody was immobilized on the fiber probe surface. After the *S. aureus* strains were bound to the antibody, the mouse monoclonal anti-*S. aureus* antibody was bound to the captured *S. aureus* to form a sandwich structure. Then, the biotin-labeled goat-anti-mouse IgG antibody bound to the detecting antibody. Finally, avidin-labeled QDs were bound by biotin to receive signals, and LOD was reported as 1 × 10^3^ CFU/mL for *S. aureus*. The selectivity of the fiberoptic immunofluorescent sensor against *S. aureus* was tested using other pathogenic bacterial strains such as *Klebsiella pneumoniae*, *P. aeruginosa*, *Acinetobacter baumannii*, and *E. coli* [119].

Wu and coworkers prepared a fluorescent probe for the determination of *E. coli* by modifying the ZnSe/ZnS QDs with 3-mercaptopropionic acid (MPA). A fluorescence examination was performed, and it was determined that the peak intensity increased with increasing cell number in the range of 10^1^–10^8^ CFU/mL with a LOD value of 10^1^ CFU/mL [120]. 

**Figure 3:**
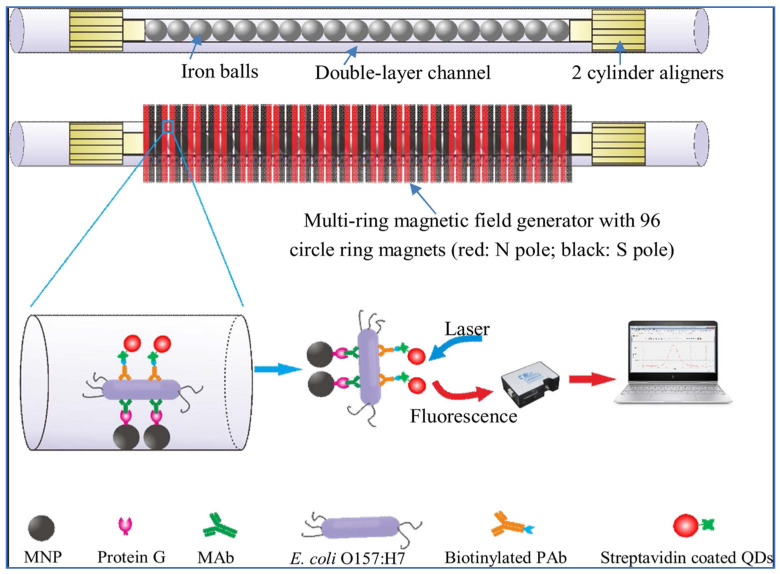
Schematic presentation of the ultrasensitive fluorescent sensing system containing bilayer channels with MNPs and QDs. Reproduced with permission from [121].

Xue et al. prepared a QDs-based fluorescent sensor for the detection of *E. coli* using magnetic separation with a double-layer channel. The schematic illustration of a fluorescent biosensor using MNPs and QDs bilayer channels is given in Figure 3. The double-layer magnetic field generator consists of 96 ring magnets. The red rings, which are among the magnets on the outside of the channel, represent the North (N) pole, and the black rings the South (S) pole. There are 1.0 mm diameter iron balls in the inner capillary of the double-layered channel. The second layer of the capillary is designed to pass the bacterial sample and reagents. The iron balls were magnetized by the magnetic field generator, creating their own magnetic field so that MNPs could be captured. Magnetic particles were modified with protein G and QDs with biotinylated *E. coli* specific polyclonal antibodies (PAb). First, modified magnetic particles were injected into the double-layer magnetic channel, and then they adhered to the channel as a monolayer. Afterwards, an *E. coli*-specific monoclonal antibody (MAb) was injected into the channel and bound to protein G. When the bacterial contaminant was injected into the canal, the bacteria specifically bound, and the impurities flowed away. Finally, the modified QDs were injected into the channel, and the resultant fluorescent sensor was ready for detection. The measurements were carried out with the optical detector, and the LOD was reported as 14 CFU/mL [121].

In another study, Hao and coworkers designed a microfluidic biosensor with a fluorescent probe for the detection of *S. typhimurium*. In this microfluidic sensor system, QDs, MnO_2_ nanoflowers (NFs), and MNPs were used together. The MnO_2_-NFs were modified with amino and the QDs with carboxyl, and they subsequently formed the MnO_2_-QDs NF complex. This complex was functionalized by immobilizing PAb. Then, MAb-immobilized MNPs and *S. typhimurium* were injected into the microfluidic channel to form MNP-bacteria-QD-MnO_2_ complexes. They found an average recovery of 99.7% in chicken meat samples containing *S. typhimurium* in the concentration range of 1.0 × 10^2^ to 1.0 × 10^7^ CFU/mL [122].

#### 3.1.4. Carbon Nanomaterials

CDs, graphene oxide (GO), and carbon nanotubes (CNTs) are some of the carbon nanomaterials. In addition to their advantages, such as their preparation by green synthesis, low toxicity, water solubility, and biocompatibility, they have been frequently applied in optical sensor platforms with high fluorescence stability due to their optical features [123].

**Figure 4:**
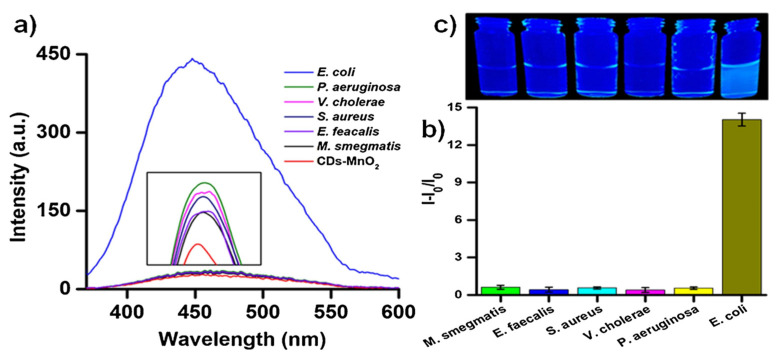
(**a**) The fluorescence output of the sensing assay indicates the selectivity. (**b**) Fluorescence output of the selectivity experiment obtained against competitive bacterial strains in a bar diagram. (**c**) Blue fluorescence response of the sensing assay. Reproduced with permission from [124].

Hiremath et al. developed a fluorescent detection system with carbon dots (CDs) and MnO_2_ for the rapid detection of *E. coli.* This system is based on an enzymatic redox reaction. This reaction was provided with a p-benzoquinone and hydroquinone redox couple. If CDs-MnO_2_ is conjugated, it does not show the fluorescence characteristic of CDs. Enzymatic reactions during the respiration of *E. coli* convert p-benzoquinone to hydroquinone by two-electron reduction, and CDs are released. Figure 4a indicates the fluorescence output of the proposed sensing platform. The LOD was found to be 50 CFU/mL. The real sample experiments were performed in rainwater, apple juice, and potato pulp. The selectivity of the fluorescent sensor was clarified by evaluating the fluorescence obtained from the proposed sensing using five different bacterial strains at a concentration of 5.0 × 10^8^ CFU/mL (Figure 4b). It can be concluded that the sensor shows high selectivity for *E. coli*. As a result, blue fluorescence was observed in the aqueous solution (Figure 4c) [124]. 

Hassan and coworkers reported the fluorescent lateral flow immunoassay (FLFIA) with GO and QDs for the detection of *E. coli* O157:H7 in minced beef and river water. The detection strip has a control line consisting of only QDs and a test line consisting of antibody-QD complexes. They prepared the test line by binding biotinylated anti-*E. coli* O157:H7 antibodies to streptavidin-conjugated CdSe@ZnS QDs. When the solution containing the microorganism is dropped on the test strip, *E. coli* binds to the antibody in the test line. GO is then added. The principle of the test is that GO extinguishes the fluorescence of QDs when combined with QDs. *E. coli* interrupts the energy flow between the QDs and the GO, so the glow continues. Consequently, LOD values were reported as 178 and 133 CFU/mL for minced meat and for river water samples, respectively [125]. 

Ondera and Hamme reported a SERS-based label-free detection method using carbon nanotubes attached to single-walled gold nanopopcorns (AuNP@f3-SWCNTs) for the detection of *E. coli.* They immobilized monoclonal anti *E. coli* antibodies to AuNP@f 3-SWCNTs and LOD was reported as 1 × 10^2^ CFU/mL for *E. coli* 49979 in aqueous solution [126].

### 3.2. Molecular Imprinting-Based Optical Sensing

Molecular imprinting is the process of creating specific recognition sites for the template molecule in a polymer. MIP-based synthetic sensors have advantages over natural/biological receptors, as they have properties such as high affinity, specificity, robustness, and low cost of production. The number of scientific publications describing MIP-based sensors has increased with the development of both polymer science and nanotechnology in recent years. MIP-based sensors have been used in medicine, biotechnology, and forensic science for diagnosing different kinds of molecules [44]. 

The molecule of interest is mixed with monomers, and monomers form a network around each target molecule in a typical process for MIP preparation. Then, the target molecule is removed, and polymeric cavities compatible with the target molecule are formed. MIPs conform to the target molecule geometrically and in terms of charge distribution. Because MIPs require relatively little time to prepare and synthetic monomers are inexpensive, the preparation of MIPs are low-cost, and Tan remains stable for a long time even under harsh conditions. Thus, the obtained MIPs are able to catch the target molecule in a complex environment. The high affinity and selectivity properties of MIPs are similar to those of natural receptors, and these properties make them useful and reliable in sensor-based assays. MIPs show superior properties to natural biomolecules due to their improved stability and simplicity of preparation [127,128]. MIPs have been prepared for many molecules and biological structures, such as nucleic acids [129], human cells [130], viruses [131], bacteria [132], drugs, and inorganic ions [133]. MIP-based sensor assays create an optical or electrochemical signal when they bind to the target molecule. Furthermore, MIP-based sensor assays are becoming more common, and the detection of target molecules in low concentrations provides advantages for diagnostic testing in real biological samples. 

Optical MIP sensors can be evaluated in two categories: affinity-based MIP sensors and optoelectronic MIP sensors. Affinity-based MIP sensors are based on the monitoring of optical changes caused by the binding of target molecules to the MIP surface of a SPR. Refractive index, fluorescence, or optical absorbance are natural optical properties that can be used to detect analytes in an affinity-based MIP sensor approach [134]. Optoelectronic MIP sensors use molecular reporters capable of producing an optical response according to the concentration of the target molecule. In addition to being thermally and photochemically stable, molecular reporters have to sense changes in their environment and respond to the presence of target molecules. High quantum yields and high molecular absorption are necessary for reporter monomers with optical properties [135]. Affinity and optoelectronic MIP sensors have become prominent approaches to designing advanced and highly accurate sensors in recent years. Nano-based materials such as carbon dots, carbon nanotubes, nanoparticles, and graphene oxide can be used to enhance the sensitivity of MIP-based sensors. Although it is a challenging process for MIP-based electrochemical and optical sensors to commercialize and take place in the biotechnology market, including the medical diagnosis field, studies in this field continue rapidly.

Fluorescence optical sensors have become advantageous among optical techniques due to their simplicity and low detection limits. MIP technology provides reproducibility, high sensitivity, and selectivity and has significant potential as an alternative to enzyme-based approaches. CDs and QDs can be used to develop MIP based sensor surfaces. CDs have unique photoluminescence properties and have great potential in biological applications. Furthermore, they are biocompatible and show chemical stability. QDs have wide absorption spectra and narrow emission, and they are also useful to be used with MIP-based sensors for pharmaceutical, medical, and environmental analysis. Surface modified QDs are able to interact with MIPs and can be used for the optical detection of many molecules. MIP-based QD sensors have advantages over immunosensors as they are chemically stable. In addition, surfaces, including NPs, are used to create MIP-based sensors, which are able to detect small molecules and are useful in designing selective sensors for environmental and pharmaceutical analysis [136].

Traditional culturing methods for the detection of microorganisms are time-consuming and require a lot of laboratory equipment. Sensor technologies provide quicker and easier methods for the identification of pathogenic microorganisms. MIP-based sensors were applied for identification and/or detection of microorganisms in the literature. In our research group, some microbial pathogens were detected with imprinting-based optical sensing. In this regard, MAH (histidine containing specific monomer) was used as a metal (Cu II)-complexing ligand. Therefore, functionalized sensing surfaces have specific recognition sites to create selectivity for the amino acids present on the bacterial cell wall. Besides complex-bound divalent heavy metal ions can easily interact with the -OH groups found on the bacterial cell wall. Representative examples from our research group are mentioned below [41].

Denizli and his colleagues developed a SPR sensor by creating a microcontact imprinted sensor for *Salmonella paratyphi* detection. *S. paratyphi* is one of the major pathogenic bacteria causing foodborne diseases. They utilized N-methacryloyl-L-histidine methyl ester (MAH) as the functional monomer to prepare it. Ellipsometry and scanning electron microscopy were used to characterize the microcontact *S. paratyphi* imprinted SPR chip. *S. paratyphi* detection was performed in the range of 2.5 × 10^6^–15 × 10^6^ CFU/mL bacteria concentration, and the LOD value was found to be 1.4 × 10^6^ CFU/mL. The specific selectivity of microcontact *S. paratyphi* imprinted SPR biosensor was verified against competing bacterial strains; *E. coli*, *S. aureus*, and *B. subtilis*. Thus, a microcontact *S. paratyphi* imprinted SPR sensor has great potential to be used for detecting *S. paratyphi* in contaminated food or water [41]. In another study, Idil and co-workers developed biomimetic sensors for *S. aureus* using a whole-cell microcontact imprinting approach. *S. aureus* imprinted SPR surfaces were prepared using MAH as the functional monomer by UV polymerization. The detection limit was reported as 1.5 × 10^3^ CFU/mL. Selectivity studies were performed with *B. subtilis*, *E. coli*, and *S. paratyphi* bacterial strains in order to verify the selectivity of the SPR sensor. The sensor responses obtained were negligible when compared to those from *S. aureus*. In addition, *S. aureus* strains were spiked into milk samples, and successful detection of the *S. aureus* in a real sample was performed [137]. In another study, Özgür et al. prepared *E. coli* receptors using the microcontact imprinting technique. They achieved label-free detection of *E. coli* cells on the SPR sensor in urine mimics and aqueous solutions. They used MAH as the functional monomer and utilized Ag nanoparticles in order to obtain low detection limits. Atomic force microscope (AFM), scanning electron microscope (SEM), ellipsometer, and contact angle measurements were used to characterize polymeric film. LOD was found to be 0.57 CFU/mL. As *E. coli* causes urinary tract infection, *E. coli* detection in urine mimic was successfully performed to evaluate the applicability of a microcontact *E. coli* imprinted SPR chip in a real biological sample [138]. 

Denizli and his colleagues developed a SPR nanosensor by using surface imprinted Au nanoparticles in order to detect *E. coli* cells. They utilized copper ions and Au nanoparticles to obtain cavities compatible with *E. coli* cells on the surface of the SPR nanosensor. AFM, ellipsometry, and contact angle measurement were used to characterize the *E. coli* imprinted SPR nanosensor. They obtained a very low detection limit of 1 CFU/mL by combining Au nanoparticles with molecular imprinting techniques. The selectivity of the SPR nanosensor was evaluated using *S. aureus*, *K. pneumoniae*, and *P. aeruginosa*. Moreover, the efficiency of the SPR nanosensor was tested on an artificial urine sample. In conclusion, the developed SPR nanosystem provides fast, sensitive, effective, and real-time detection of *E. coli* cells [139]. Çimen et al. prepared an endotoxin-imprinted poly(hydroxyethyl methacrylate-N-methacryloyl-(L)-histidine methyl ester) based nanofilm on the SPR chip surface by UV polymerization. Sensing studies were performed in the range of 0.5–100 ng/mL endotoxin concentration. The LOD value was found to be 0.023 ng/mL. Cholesterol and hemoglobin were used as competing molecules to evaluate the selectivity of the endotoxin imprinted SPR sensor [140]. Ochratoxin A (OTA) is one of the mycotoxins that frequently contaminates food products. Denizli and co-workers designed a molecular imprinting-based SPR sensor to detect OTA in dried figs. First, the gold chip surface was functionalized with allyl mercaptan, and then a polymerization solution was added to the modified chip surface. The polymerization solution contains OTA as the template molecule, N-methacryl-(L)-phenylalanine (MAPA) as the functional monomer, 2-Hydroxyethyl methacrylate (HEMA) as the monomer, Ethylene glycol dimethylacrylate (EGDMA) as the crosslinker and Azobisisobutyronitrile (AIBN) as the initiator. The recognition of OTA by the molecularly imprinted optical sensor has been reported to be based on the hydrophobic interactions between OTA and MAPA. The LOD was reported as 0.028 ng/mL [141]. In the same research group, a mycotoxin, aflatoxin B1 (AFB1), was detected in corn and peanuts with a molecularly imprinted AuNPs-coated SPR sensor. The LOD was found to be 1.04 pg/mL. The selectivity of the AFB1 imprinted sensor was verified by evaluating the sensing responses against Aflatoxin B2, aflatoxin M1, OTA, and citrinin at an initial concentration of 0.1 ng/mL, and the relative selectivity coefficients (k’) of the AFB1 imprinted SPR sensor were calculated as 3.81, 2.89, 12.72, and 15.14, respectively [142]. Turkmen and his colleagues presented an alternative approach for bacterial growth control. *Pseudomonas* sp. is a major cause of food contamination, especially in meat products. Thus, fast and reliable detection of *Pseudomonas* sp. is important. They prepared *Pseudomonas* sp. imprinted nanofilm on the SPR sensor surface and used functional monomers MAH and Cu ions to develop a microcontact imprinted nanofilm surface. The LOD value was reported as 0.5 × 10^2^ CFU/mL. Selectivity studies were performed with *S. aureus*, *S. paratyphi*, and *E. coli* cells, and the SPR sensor showed high selectivity for *Pseudomonas* sp. cells. *Pseudomonas*-microcontact imprinted SPR sensors were shown to be used five times efficiently [143]. 

Bezdekova and co-workers constructed a detection method based on molecular imprinting and fluorescent microscopy for the detection of *S. aureus* found in milk and rice samples. Dopamine was applied as a functional monomer. After the extraction of the target bacterial strains, they were imprinted on the surface of the magnetic particles (MIPs). The proposed sensing method was reported as a good candidate for bacterial food control. The LOD value was found to be 1 × 10^3^ CFU/mL, conforming to the limit set in European Union legislation for microbial control of food [144]. 

Zhao et al., prepared *L. monocytogenes* imprinted polymer with CdTe QDs by oil-in-water Pickering emulsion polymerization. The authors utilized chitosan to create recognition sites compatible with *L. monocytogenes* on the surface of microspheres loaded with CdTe QDs. SEM characterization showed that the target bacteria cells adsorbed effectively on the imprinted polymers. The authors achieved qualitative detection of *L. monocytogenes* cells in food samples such as pork and milk. A developed fluorescence sensor provided effective, reliable, and fast detection of *L. monocytogenes* cells [145]. 

Guo et al., presented a novel method for *S. aureus* detection. Since *S. aureus* can cause food contamination, effective identification is needed. Guo and his colleagues combined *S. aureus* imprinted polydimethylsiloxane film for specific recognition with a fluorescence resonance energy transfer platform for fluorescence sensing. They used the stamp imprinting method to get cavities complementary to *S. aureus*. They also developed turn-on fluorescence, taking advantage of the electrostatic interaction between copper clusters and dopamine-stabilized gold nanoparticles. They obtained a LOD of 11.12 CFU/mL with this fast, sensitive, and label-free method [146]. 

### 3.3. Hydrogel-Based Optical Sensing

Hydrogels are hydrophilic, flexible, and porous materials that can be prepared as natural or synthetic polymers. The porous and reticulated structures of hydrogels have applications in different fields, such as tissue engineering, drug delivery, and detection platforms. They can be easily incorporated with the particles due to their porous structure [147]. Hydrogel-based optical sensing platforms can be prepared by using fluorescent nanoparticles and substrates of enzymes. This way, color change occurs in the presence of an enzyme-substrate reaction. They can also be synthesized as super-flexible materials [148]. In recent years, it has been applied in the development of wearable electronics for motion detection and biomedical monitoring due to its flexible and conductive structures [149,150]. Another advantage of hydrogels is that they can be easily functionalized with functional molecules and can respond to stimuli. Thus, it becomes easier to prepare hydrogels as target-specific materials. Apart from these, they can be designed to detect chemical (pH, concentration, type of molecule) as well as physical (temperature, electric or magnetic field, light, and pressure) changes [151,152]. Hydrogels have been widely preferred in many different sensor applications due to their detectable chemical, electrical, and optical responses to external stimuli [153]. From the point of view of biocompatibility, biocompatible hydrogels have also contributed to the emergence of new optical sensor technologies based on in vivo measurements of physiological parameters [154]. Some examples from related literature were given for microbial contaminant sensing applications using hydrogel-based optical sensors in recent years.

Gunda et al. designed agarose hydrogels for the enzymatic reaction-based detection of *E. coli* from contaminated water. They impregnated substrates of enzymes found in *E. coli*, such as bacteria protein extraction reagent (B-PER) and 6-chloro-3-indolyl-β-D-galactopyranoside (Red-Gal), into the hydrogel solution. *E. coli* was quantitatively determined by the degradation and color change of the substrates in the presence of enzymes [155].

Junk et al. synthesized poly(N-isopropylacrylamide) (pNIPAM) based photonic crystal hydrogel films to detect metabolic products such as alcohol and succinic acid using *Zymomonas mobilis* and *Actinobacillus succinogenes* strains. Hydrogel films were synthesized onto the surface of a 96 well plate. First, the wells were functionalized with methacrylate groups. Hydrogels were synthesized by UV polymerization by adding negatively charged polystyrene (PS) particles to the p(NIPAM) based polymer solution. The susceptibility of photonic crystal hydrogel films to various short-chain alcohols and organic acids was determined using a microplate reader [156].

**Figure 5:**
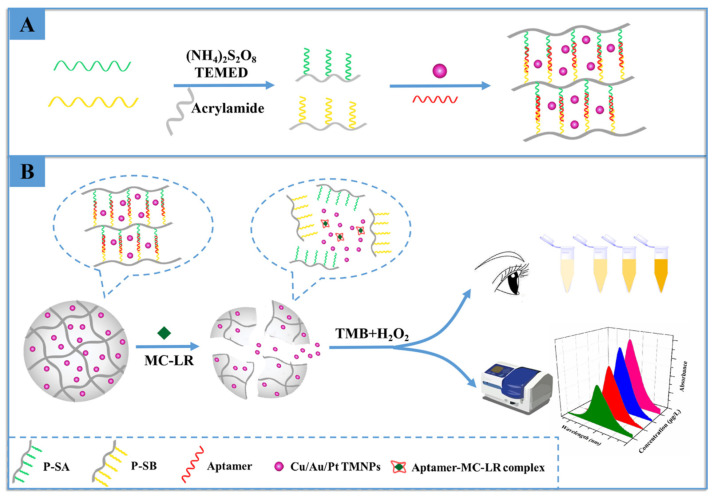
Schematic illustration of the colorimetric biosensor for the detection of MC-LR based on the Cu/Au/Pt TNs-encapsulated DNA hydrogel. (**A**) The preparation of the Cu/Au/Pt TNs-encapsulated DNA hydrogel, (**B**) The analysis step for Microcystin-LR. Reproduced with permission from [158].

Hydrogels hybridized with DNA/aptamers have been frequently used in recent years for the specific detection of biotoxins. Microcystin-LR is produced by cyanobacteria, and according to the World Health Organization, the highest amount that can be found in drinking water is 1.0 μg/L [157]. Wu et al. prepared a DNA hydrogel with Cu/Au/Pt trimetallic nanoparticles (TNs) encapsulated for the sensitive detection of microcystin-LR [158]. The DNA-hydrogel was designed to recognize MC-LR (Figure 5). Hydrogels were synthesized on polyacrylamide using APS/TEMED as the initiator/activator pair. Here, MC-LR aptamers are used as crosslinkers. As seen in Figure 5, strips were designed, and then A and B strips were designated to complement two regions of the MC-LR aptamer. By connecting these strands to the hydrogel, they prepared polymer strips A (P-SA) and B (P-SB). Cu/Au/Pt TNs were embedded in the hydrogel. In the presence of MC-LR, aptamers act as crosslinkers bound to MC-LR. When the MC-LR complex was formed with the specific aptamer, the hydrogels were depredated, and Cu/Au/Pt TNs were released. The released Cu/Au/Pt TNs catalyzed the TMB, and H_2_O_2_ was added to the medium for the catalytic reaction. It results in color changes depending on the change in MC-LR concentration.

Another application of the DNA aptamer cross-linked hydrogel optical sensor was developed by Ma et al. for the determination of Aflatoxin B1 (AFB1). They loaded AuNP on hydrogels for optical detection. AFB1 in the medium bound competitively with the specific aptamer, and AuNP in hydrogels was revealed in red. AuNP-loaded hydrogels had a color indicator in the 0.25–40 µM range to detect AFB 1 [159].

## 4. Conclusions and Future Perspectives

Potential microbial contaminants in food, water, and the environment have become a problem of special importance worldwide. Culture-independent methods enable the development of much more rapid, specific, sensitive, reusable, and convenient approaches for the detection of causative microbial strains. It is noteworthy to point out that discrimination between dead and live microorganisms still remains a challenge, and non-culturable ones can be overlooked. One of the most important points to be made is that commercializing analytical tools makes them applicable in the fields of food, water, and the environment to quantify microbial contaminants. Among optical sensing platforms, colorimetric and fluorescence-based platforms have been introduced as commercialized sensing devices. In this respect, the high cost of the establishment of these tools puts restrictions on their applications for food safety and water quality. Optical sensing has complexity in terms of the sources required for sensing and monitoring, such as light, fluorometers, and spectrometers. Another limitation is using denaturable ligand molecules with heat, including enzymes and antibodies; therefore, the sterilization problem limits the ability to create reusable sensing systems. From the point of view of aptamers, they are advantageous over antibodies due to their improved stability, designable properties, and reusability for the fabrication of sensors. On the other hand, they can be easily degraded in biological media because they can interact with biomolecules.

Molecular imprinting technology is a promising approach that aims to overcome these limitations and difficulties in microorganism detection in a simple way. MIPs have been introduced as unique polymeric materials for the development of standardized, stable, and practical detection devices that have been used in different applications. It is noteworthy to indicate that the applicability of MIPs has been proven with many reports, and they will shed light on future initiatives regarding sensor applications. The methodologies are still needed to overcome challenges in terms of complexities in the discrimination of microbial strains having similar size and shape. Recent advances show that paper-based colorimetric approaches are successful in improving POC devices and have gained much more interest due to their facile, low-cost preparation and the opportunity to obtain signals with the naked eye. 

Nanomaterials have been easily integrated into sensing systems due to their properties of rapid, sensitive, and easy detection, along with enabling the construction of portable devices. Their combination with sensing and potential applicability have been proven; however, their usage in the medical field is still required to be clarified. 

It is considered that it will be possible to create cost-effective, accurate, multiple-functionalized, miniaturized, and integrated platforms for the detection of microbial contaminants by developing and commercializing sensor devices with different, much more sensitive, selective, accurate, and successful approaches to be used in daily life. The text continues here. SERS, interferometric, and plasmonic sensing systems are still improving. Small molecules are much more appropriate to be detectable by plasmonic sensors; however, the larger dimensions of microorganisms and the smaller penetration depths of the electromagnetic field limit the detection stage. The low refractive index restricts the detection in aqueous solutions and the suitability and attainability of the recognition elements in the microbial target regions. In light of these, optical sensing needs to be more developed for the fabrication of POCs. There are some drawbacks to using them as clinical diagnostic tools, and some key points are supposed to be appealed to in future strategies. 

## Data Availability

No new data were created.

## Abbreviations

AAEM	Acetoacetoxy ethyl methacrylate
AFB 1	Aflatoxin B 1
AFM	Atomic force microscope
AIBN	Azobisisobutyronitrile
AuNPs	Gold nanoparticles
cApt	Complementary aptamer
CDs	Carbon dots
CFU	Colony forming unit
DSNB	5,5′-dithiobis succinimidyl-2-nitrobenzoate
EGDMA	Ethylene glycol dimethylacrylate
ELISA	Enzyme-linked immunosorbent assay
FMSs	Polyclonal antibody-modified microspheres
FRET	Fluorescence resonance energy transfer
GNRs	Gold nanorods
GO	Graphene oxide
HEMA	2-Hydroxyethyl methacrylate
LAMP	loop-mediated isothermal amplification
LOD	Limit of detection
LOQ	The limit of quantification
LSPR	Localized surface plasmon resonance
MAH	N-methacryloyl-L-histidine methyl ester
MAPA	N-methacryl-(L)-phenylalanine
MBA	4-mercapto benzoic acid
MC-LR	Microcystin-LR
MIP	Molecularly imprinted polymer
MMA	Methyl methacrylate
MNPs	Magnetic nanoparticles
MPA	3-mercaptopropionic acid
NBs	Nanobones
NPs	Nanoparticles
OTA	Ochratoxin A
OTS	Octadecyltrichlorosilanes
PFMNPs	Polymeric magnetic nanoparticles
PLS-DA	Partial least squares discriminant analysis
pNIPAM	poly(N-isopropylacrylamide)
POC	Point-of-care
QDs	Quantum dots
rGO	Reduced graphene oxide
SEM	Scanning electron microscope
SERS	Surface enhanced Raman scattering
SPR	Surface plasmon resonance
St	Styrene
TMB	3,5,3′,5′ tetramethyl benzidine
UFOB	U-bent fiber optic probe

## References

[1] Serwecinska L. (2020). Antimicrobials and antibiotic-resistant bacteria. Water.

[2] Doron S. (2020). Bacterial Infections: Overview. Int. Encycl. Public Health.

[3] Elbashir S., Parveen S., Schwarz J., Rippen T., Jahncke M., DePaola A. (2018). Seafood pathogens and information on antimicrobial resistance: A review. Food Microbiol..

[4] Picciotti U., Dalbon V.A., Ciancio A., Colagiero M., Cozzi G., De Bellis L., Finetti-sialer M.M., Greco D., Ippolito A., Lahbib N. (2023). “Ectomosphere”: Insects and Microorganism Interactions. Microorganisms.

[5] Geletu U.S., Usmael M.A., Ibrahim A.M. (2022). Isolation, identification, and susceptibility profile of *E. coli*, *Salmonella*, and *S. aureus* in dairy farm and their public health implication in Central Ethiopia. Vet. Med. Int..

[6] Tian L., Sun Z., Zhang Z. (2018). Antimicrobial resistance of pathogens causing nosocomial bloodstream infection in Hubei Province, China, from 2014 to 2016: A multicenter retrospective study. BMC Public Health.

[7] Fusco V., Abriouel H., Benomar N., Kabisch J., Chieffi D., Cho G.-S., Franz C.M.A.P. (2018). Opportunistic Food-Borne Pathogens. Food Safety and Preservation.

[8] Llor C., Bjerrum L. (2014). Antimicrobial resistance: Risk associated with antibiotic overuse and initiatives to reduce the problem. Ther. Adv. Drug Saf..

[9] Sekoni K.F., Oreagba I.A., Oladoja F.A. (2022). Antibiotic utilization study in a teaching hospital in Nigeria. JAC-Antimicrob. Resist..

[10] Maragakis L.L., Perencevich E.N., Cosgrove S.E. (2014). Clinical and economic burden of antimicrobial resistance. Expert Rev. Anti-Infect. Ther..

[11] Murray C.J., Ikuta K.S., Sharara F., Swetschinski L., Robles Aguilar G., Gray A., Han C., Bisignano C., Rao P., Wool E. (2022). Global burden of bacterial antimicrobial resistance in 2019: A systematic analysis. Lancet.

[12] Dadgostar P. (2019). Antimicrobial resistance: Implications and costs. Infect. Drug Resist..

[13] Gizaw Z. (2019). Public health risks related to food safety issues in the food market: A systematic literature review. Environ. Health Prev. Med..

[14] Bhalla T.C., Monika, Sheetal, Savitri (2019). International Laws and Food-Borne Illness. Food Safety and Human Health.

[15] Todd E.C.D. (2014). Foodborne diseases: Overview of biological hazards and foodborne diseases. Encycl. Food Saf..

[16] Bantawa K., Sah S.N., Subba Limbu D., Subba P., Ghimire A. (2019). Antibiotic resistance patterns of *Staphylococcus aureus*, *Escherichia coli*, *Salmonella*, *Shigella* and *Vibrio* isolated from chicken, pork, buffalo and goat meat in eastern Nepal. BMC Res. Notes.

[17] Ramírez-Castillo F.Y., Loera-Muro A., Jacques M., Garneau P., Avelar-González F.J., Harel J., Guerrero-Barrera A.L. (2015). Waterborne pathogens: Detection methods and challenges. Pathogens.

[18] Seboka B.T., Hailegebreal S., Yehualashet D.E., Kabthymer R.H., Negas B., Kanno G.G., Tesfa G.A., Yasmin F. (2022). Methods used in the spatial analysis of diarrhea: A protocol for a systematic review. Med. Case Rep. Study Protoc..

[19] Ahmed J., Wong L.P., Chua Y.P., Channa N., Mahar R.B., Yasmin A., Vanderslice J.A., Garn J.V. (2020). Quantitative microbial risk assessment of drinking water quality to predict the risk of waterborne diseases in primary-school children. Int. J. Environ. Res. Public Health.

[20] Nnachi R.C., Sui N., Ke B., Luo Z., Bhalla N., He D., Yang Z. (2022). Biosensors for rapid detection of bacterial pathogens in water, food and environment. Environ. Int..

[21] Lobnik A., Turel M., Korent S. (2012). Optical chemical sensors: Design and applications. Advances in Chemical Sensors.

[22] Mocan T., Matea C.T., Pop T., Mosteanu O., Buzoianu A.D., Puia C., Iancu C., Mocan L. (2017). Development of nanoparticle-based optical sensors for pathogenic bacterial detection. J. Nanobiotechnol..

[23] Singh S., Kumar V., Dhanjal D.S., Datta S., Prasad R., Singh J. (2020). Biological biosensors for monitoring and diagnosis. Microbial Biotechnology: Basic Research and Applications.

[24] Poltronieri P., Mezzolla V., Primiceri E., Maruccio G. (2014). Biosensors for the detection of food pathogens. Foods.

[25] Pebdeni A.B., Roshani A., Mirsadoughi E., Behzadifar S., Hosseini M. (2022). Recent advances in optical biosensors for specific detection of *E. coli* bacteria in food and water. Food Control.

[26] Ji J., Schanzle J.A., Tabacco M.B. (2004). Real-time detection of bacterial contamination in dynamic aqueous environments using optical sensors. Anal. Chem..

[27] Wang D., Sheng B., Peng L., Huang Y., Ni Z. (2019). Flexible and optical fiber sensors composited by graphene and PDMS for motion detection. Polymers.

[28] Yu T., Zheng Z., Guo K., Zhao J., Dai Q., Li H., Pons-Moll G., Liu Y. DoubleFusion: Real-Time Capture of Human Performances with Inner Body Shapes From a Single Depth Sensor. Proceedings of the IEEE Conference on Computer Vision and Pattern Recognition (CVPR).

[29] Zhou Z., Zhang Y., Guo M., Huang K., Xu W. (2020). Ultrasensitive magnetic DNAzyme-copper nanoclusters fluorescent biosensor with triple amplification for the visual detection of *E. coli* O157:H7. Biosens. Bioelectron..

[30] Masdor N.A., Altintas Z., Shukor M.Y., Tothill I.E. (2019). Subtractive inhibition assay for the detection of Campylobacter jejuni in chicken samples using surface plasmon resonance. Sci. Rep..

[31] Raghu H.V., Kumar N. (2020). Rapid Detection of Listeria monocytogenes in Milk by Surface Plasmon Resonance Using Wheat Germ Agglutinin. Food Anal. Methods.

[32] Khan S.A., DeGrasse J.A., Yakes B.J., Croley T.R. (2015). Rapid and sensitive detection of cholera toxin using gold nanoparticle-based simple colorimetric and dynamic light scattering assay. Anal. Chim. Acta.

[33] Torun Ö., Hakki Boyaci I., Temür E., Tamer U. (2012). Comparison of sensing strategies in SPR biosensor for rapid and sensitive enumeration of bacteria. Biosens. Bioelectron..

[34] Guven B., Basaran-Akgul N., Temur E., Tamer U., Boyaci I.H. (2011). SERS-based sandwich immunoassay using antibody coated magnetic nanoparticles for Escherichia coli enumeration. Analyst.

[35] Wang P., Sun H., Yang W., Fang Y. (2022). Optical methods for label-free detection of bacteria. Biosensors.

[36] Ferreira M.D.P., Yamada-Ogatta S.F., Teixeira Tarley C.R. (2023). Electrochemical and Bioelectrochemical Sensing Platforms for Diagnostics of COVID-19. Biosensors.

[37] Yang S.Z., Liu Q.A., Liu Y.L., Weng G.J., Zhu J., Li J.J. (2021). Recent progress in the optical detection of pathogenic bacteria based on noble metal nanoparticles. Microchim. Acta.

[38] Ebralidze I.I., Laschuk N.O., Poisson J., Zenkina O.V. (2019). Colorimetric Sensors and Sensor Arrays. Nanomater. Des. Sens. Appl..

[39] Huang Q.D., Lv C.H., Yuan X.L., He M., Lai J.P., Sun H. (2021). A novel fluorescent optical fiber sensor for highly selective detection of antibiotic ciprofloxacin based on replaceable molecularly imprinted nanoparticles composite hydrogel detector. Sens. Actuators B Chem..

[40] Akkin T., Dave D.P., Milner T.E., Rylander H.G. (2002). Interferometric fiber-based optical biosensor to measure ultra-small changes in refractive index. Optical Fibers and Sensors for Medical Applications II, Proceedings of the International Symposium on Biomedical Optics, San Jose, CA, USA, 22–23 January 2002.

[41] Perçin I., Idil N., Bakhshpour M., Yılmaz E., Mattiasson B., Denizli A. (2017). Microcontact imprinted plasmonic nanosensors: Powerful tools in the detection of salmonella paratyphi. Sensors.

[42] Ho C.S., Jean N., Hogan C.A., Blackmon L., Jeffrey S.S., Holodniy M., Banaei N., Saleh A.A.E., Ermon S., Dionne J. (2019). Rapid identification of pathogenic bacteria using Raman spectroscopy and deep learning. Nat. Commun..

[43] Chattopadhyay S., Sabharwal P.K., Jain S., Kaur A., Singh H. (2019). Functionalized polymeric magnetic nanoparticle assisted SERS immunosensor for the sensitive detection of *S. typhimurium*. Anal. Chim. Acta.

[44] Ahmad O.S., Bedwell T.S., Esen C., Garcia-Cruz A., Piletsky S.A. (2019). Molecularly imprinted polymers in electrochemical and optical sensors. Trends Biotechnol..

[45] Ahmed A., Rushworth J.V., Hirst N.A., Millner P.A. (2014). Biosensors for whole-cell bacterial detection. Clin. Microbiol. Rev..

[46] Chen Y.T., Lee Y.C., Lai Y.H., Lim J.C., Huang N.T., Lin C.T., Huang J.J. (2020). Review of integrated optical biosensors for point-of-care applications. Biosensors.

[47] Mauriz E. (2020). Clinical applications of visual plasmonic colorimetric sensing. Sensors.

[48] Kandasamy K., Jannatin M., Chen Y.C. (2021). Rapid detection of pathogenic bacteria by the naked eye. Biosensors.

[49] Liu G., Lu M., Huang X., Li T., Xu D. (2018). Application of gold-nanoparticle colorimetric sensing to rapid food safety screening. Sensors.

[50] Yu L., Li N. (2019). Noble metal nanoparticles-based colorimetric biosensor for visual quantification: A mini review. Chemosensors.

[51] Wu S., Duan N., Qiu Y., Li J., Wang Z. (2017). Colorimetric aptasensor for the detection of Salmonella enterica serovar typhimurium using ZnFe_2_O_4_-reduced graphene oxide nanostructures as an effective peroxidase mimetics. Int. J. Food Microbiol..

[52] Suaifan G.A.R.Y., Alhogail S., Zourob M. (2017). Rapid and low-cost biosensor for the detection of *Staphylococcus aureus*. Biosens. Bioelectron..

[53] Kaur B., Kumar S., Kaushik B.K. (2023). Trends, challenges, and advances in optical sensing for pathogenic bacteria detection (PathoBactD). Biosens. Bioelectron. X.

[54] Yoon S.A., Park S.Y., Cha Y., Gopala L., Lee M.H. (2021). Strategies of Detecting Bacteria Using Fluorescence-Based Dyes. Front. Chem..

[55] Elahi N., Kamali M., Baghersad M.H., Amini B. (2019). A fluorescence Nano-biosensors immobilization on Iron (MNPs) and gold (AuNPs) nanoparticles for detection of *Shigella* spp.. Mater. Sci. Eng. C.

[56] Pathak A., Venugopal P., Nair B.G., Suneesh P.V., Satheesh Babu T.G. (2020). Facile pH-sensitive optical detection of pathogenic bacteria and cell imaging using multi-emissive nitrogen-doped carbon dots. Microchem. J..

[57] Bahari D., Babamiri B., Salimi A., Salimizand H. (2021). Ratiometric fluorescence resonance energy transfer aptasensor for highly sensitive and selective detection of Acinetobacter baumannii bacteria in urine sample using carbon dots as optical nanoprobes. Talanta.

[58] Turner A.P.F., Zourob M., Turner A. (2008). Principles of Bacterial Detection: Biosensors, Recognition Receptors and Microsystems.

[59] Yang F., Chang T.L., Liu T., Wu D., Du H., Liang J., Tian F. (2019). Label-free detection of Staphylococcus aureus bacteria using long-period fiber gratings with functional polyelectrolyte coatings. Biosens. Bioelectron..

[60] Janik M., Brzozowska E., Czyszczoń P., Celebańska A., Koba M., Gamian A., Bock W.J., Śmietana M. (2021). Optical fiber aptasensor for label-free bacteria detection in small volumes. Sens. Actuators B Chem..

[61] Dudak F.C., Boyaci I.H. (2009). Rapid and label-free bacteria detection by surface plasmon resonance (SPR) biosensors. Biotechnol. J..

[62] Asir S., Bakhshpour M., Unal S., Denizli A. (2022). Nanoparticle-based plasmonic devices for bacteria and virus recognition. Mod. Pract. Healthc. Issues Biomed. Instrum..

[63] Tang Y., Zeng X., Liang J. (2010). Surface plasmon resonance: An introduction to a surface spectroscopy technique. J. Chem. Educ..

[64] Wang Y., Knoll W., Dostalek J. (2012). Bacterial pathogen surface plasmon resonance biosensor advanced by long range surface plasmons and magnetic nanoparticle assays. Anal. Chem..

[65] Das P., Fatehbasharzad P., Colombo M., Fiandra L., Prosperi D. (2019). Multifunctional magnetic gold nanomaterials for cancer. Trends Biotechnol..

[66] Kaya H.O., Cetin A.E., Azimzadeh M., Topkaya S.N. (2021). Pathogen detection with electrochemical biosensors: Advantages, challenges and future perspectives. J. Electroanal. Chem..

[67] Li M., Singh R., Wang Y., Marques C., Zhang B., Kumar S. (2022). Advances in novel nanomaterial-based optical fiber biosensors—A review. Biosensors.

[68] Waswa J., Irudayaraj J., DebRoy C. (2007). Direct detection of *E. coli* O157:H7 in selected food systems by a surface plasmon resonance biosensor. LWT-Food Sci. Technol..

[69] Chemburu S., Wilkins E., Abdel-Hamid I. (2005). Detection of pathogenic bacteria in food samples using highly-dispersed carbon particles. Biosens. Bioelectron..

[70] Petryayeva E., Krull U.J. (2011). Localized surface plasmon resonance: Nanostructures, bioassays and biosensing—A review. Anal. Chim. Acta.

[71] Pellas V., Hu D., Mazouzi Y., Mimoun Y., Blanchard J., Guibert C., Salmain M., Boujday S. (2020). Gold nanorods for LSPR biosensing: Synthesis, coating by silica, and bioanalytical applications. Biosensors.

[72] Oh S.Y., Heo N.S., Bajpai V.K., Jang S.C., Ok G., Cho Y., Huh Y.S. (2019). Development of a cuvette-based LSPR sensor chip using a plasmonically active Transparent Strip. Front. Bioeng. Biotechnol..

[73] Dursun A.D., Borsa B.A., Bayramoglu G., Arica M.Y., Ozalp V.C. (2022). Surface plasmon resonance aptasensor for *Brucella* detection in milk. Talanta.

[74] Yamasaki T., Miyake S., Nakano S., Morimura H., Hirakawa Y., Nagao M., Iijima Y., Narita H., Ichiyama S. (2016). Development of a surface plasmon resonance-based immunosensor for detection of 10 major O-Antigens on shiga toxin-producing *Escherichia coli*, with a gel displacement technique to remove bound bacteria. Anal. Chem..

[75] Zhang X., Tsuji S., Kitaoka H., Kobayashi H., Tamai M., Honjoh K.I., Miyamoto T. (2017). Simultaneous detection of *Escherichia coli* O157:H7, *Salmonella enteritidis*, and *Listeria monocytogenes* at a very low level using simultaneous enrichment broth and multichannel SPR biosensor. J. Food Sci..

[76] Akimov N., Scudder J., Ye J.Y. (2021). Refinement of an open-microcavity optical biosensor for bacterial endotoxin test. Biosens. Bioelectron..

[77] Nakano S., Nagao M., Yamasaki T., Morimura H., Hama N., Iijima Y., Shinomiya H., Tanaka M., Yamamoto M., Matsumura Y. (2018). Evaluation of a surface plasmon resonance imaging-based multiplex O-antigen serogrouping for *Escherichia coli* using eleven major serotypes of Shiga-toxin-producing *E. coli*. J. Infect. Chemother..

[78] Khateb H., Klös G., Meyer R.L., Sutherland D.S. (2020). Development of a label-free LSPR-apta sensor for *Staphylococcus aureus* detection. ACS Appl. Bio Mater..

[79] Lee N., Choi S.W., Chang H.J., Chun H.S. (2018). Rapid detection of *Escherichia coli* O157:H7 in fresh lettuce based on Localized Surface Plasmon Resonance combined with immunomagnetic separation. J. Food Prot..

[80] Yaghubi F., Zeinoddini M., Saeedinia A.R., Azizi A., Samimi Nemati A. (2020). Design of Localized Surface Plasmon Resonance (LSPR) biosensor for immunodiagnostic of *E. coli* O157:H7 using gold nanoparticles conjugated to the chicken antibody. Plasmonics.

[81] Song L., Zhang L., Huang Y., Chen L., Zhang G., Shen Z., Zhang J., Xiao Z., Chen T. (2017). Amplifying the signal of localized surface plasmon resonance sensing for the sensitive detection of *Escherichia coli* O157:H7. Sci. Rep..

[82] Wang L., Liu W., Tang J., Wang J., Liu Q., Wen P., Wang M., Pan Y., Gu B., Zhang X. (2021). Applications of Raman Spectroscopy in bacterial infections: Principles, advantages, and shortcomings. Front. Microbiol..

[83] Bräuer B., Thier F., Bittermann M., Baurecht D., Lieberzeit P.A. (2022). Raman studies on surface-imprinted polymers to distinguish the polymer surface, imprints, and different bacteria. ACS Appl. Bio Mater..

[84] Liu S., Hu Q., Li C., Zhang F., Gu H., Wang X., Li S., Xue L., Madl T., Zhang Y. (2021). Wide-range, rapid, and specific identification of pathogenic bacteria by Surface-Enhanced Raman Spectroscopy. ACS Sens..

[85] Saletnik A., Saletnik B., Puchalski C. (2021). Overview of popular techniques of raman spectroscopy and their potential in the study of plant tissues. Molecules.

[86] Pérez-Jiménez A.I., Lyu D., Lu Z., Liu G., Ren B. (2020). Surface-enhanced Raman spectroscopy: Benefits, trade-offs and future developments. Chem. Sci..

[87] Jans H., Huo Q. (2023). Gold nanoparticle-enabled biological and chemical detection and analysis. Chem. Soc. Rev..

[88] Zhou X., Hu Z., Yang D., Xie S., Jiang Z., Niessner R., Haisch C., Zhou H., Sun P. (2020). Bacteria detection: From powerful SERS to its advanced compatible techniques. Adv. Sci..

[89] Thvenot D.R., Toth K., Durst R.A., Wilson G.S. (1999). Electrochemical biosensors: Recommended definitions and classification (Technical Report). Pure Appl. Chem..

[90] Sau T.K., Rogach A.L., Jäckel F., Klar T.A., Feldmann J. (2010). Properties and Applications of Colloidal Nonspherical Noble Metal Nanoparticles. Adv. Mater..

[91] Moores A., Goettmann F. (2006). The plasmon band in noble metal nanoparticles: An introduction to theory and applications. New J. Chem..

[92] Tan F., Yang Y., Xie X., Wang L., Deng K., Xia X., Yang X., Huang H. (2018). Prompting peroxidase-like activity of gold nanorod composites by localized surface plasmon resonance for fast colorimetric detection of prostate specific antigen. Analyst.

[93] Malekzad H., Sahandi Zangabad P., Mirshekari H., Karimi M., Hamblin M.R. (2017). Noble metal nanoparticles in biosensors: Recent studies and applications. Nanotechnol. Rev..

[94] Wang H., Rao H., Luo M., Xue X., Xue Z., Lu X. (2019). Noble metal nanoparticles growth-based colorimetric strategies: From monocolorimetric to multicolorimetric sensors. Coord. Chem. Rev..

[95] Sivakumar R., Dinh V.P., Lee N.Y. (2021). Ultraviolet-induced in situ gold nanoparticles for point-of-care testing of infectious diseases in loop-mediated isothermal amplification. Lab Chip.

[96] Kumar H., Kuča K., Bhatia S.K., Saini K., Kaushal A., Verma R., Bhalla T.C., Kumar D. (2020). Applications of Nanotechnology in Sensor-Based Detection of Foodborne Pathogens. Sensors.

[97] Du H., Li Z., Wang Y., Yang Q., Wu W. (2020). Nanomaterial-based optical biosensors for the detection of foodborne bacteria. Food Rev. Int..

[98] Yoo S.M., Kim D.K., Lee S.Y. (2015). Aptamer-functionalized localized surface plasmon resonance sensor for the multiplexed detection of different bacterial species. Talanta.

[99] Huang W., Zhou X., Luan Y., Cao Y., Wang N., Lu Y., Liu T., Xu W. (2020). A sensitive electrochemical sensor modified with multi-walled carbon nanotubes doped molecularly imprinted silica nanospheres for detecting chlorpyrifos. J. Sep. Sci..

[100] Bhaisare M.L., Gedda G., Khan M.S., Wu H.F. (2016). Fluorimetric detection of pathogenic bacteria using magnetic carbon dots. Anal. Chim. Acta.

[101] Morales-Narváez E., Naghdi T., Zor E., Merkoçi A. (2015). Photoluminescent lateral-flow immunoassay revealed by graphene oxide: Highly sensitive paper-based pathogen Detection. Anal. Chem..

[102] Ko Y.C., Fang H.Y., Chen D.H. (2017). Fabrication of Ag/ZnO/reduced graphene oxide nanocomposite for SERS detection and multiway killing of bacteria. J. Alloys Compd..

[103] Li Y., Yang J., Zhong T., Zhao N., Liu Q.Q., Shi H.F., Xu H.M. (2017). Fast and green synthesis of silver nanoparticles/reduced graphene oxide composite as efficient surface-enhanced Raman scattering substrate for bacteria detection. Monatshefte Chem..

[104] Duan N., Chang B., Zhang H., Wang Z., Wu S. (2016). *Salmonella typhimurium* detection using a surface-enhanced Raman scattering-based aptasensor. Int. J. Food Microbiol..

[105] Zhao Y., Li Y., Zhang P., Yan Z., Zhou Y., Du Y., Qu C., Song Y., Zhou D., Qu S. (2021). Cell-based fluorescent microsphere incorporated with carbon dots as a sensitive immunosensor for the rapid detection of *Escherichia coli* O157 in milk. Biosens. Bioelectron..

[106] Zhou S., Lu C., Li Y., Xue L., Zhao C., Tian G., Bao Y., Tang L., Lin J., Zheng J. (2020). Gold nanobones enhanced ultrasensitive Surface-Enhanced Raman Scattering aptasensor for detecting *Escherichia coli* O157:H7. ACS Sens..

[107] Wu S., Duan N., He C., Yu Q., Dai S., Wang Z. (2020). Surface-enhanced Raman spectroscopic–based aptasensor for *Shigella sonnei* using a dual-functional metal complex-ligated gold nanoparticles dimer. Colloids Surf. B Biointerfaces.

[108] Dehghani Z., Mohammadnejad J., Hosseini M., Bakhshi B., Rezayan A.H. (2020). Whole cell FRET immunosensor based on graphene oxide and graphene dot for *Campylobacter jejuni* detection. Food Chem..

[109] Hu X., Li Y., Xu Y., Gan Z., Zou X., Shi J., Huang X., Li Z., Li Y. (2021). Green one-step synthesis of carbon quantum dots from orange peel for fluorescent detection of *Escherichia coli* in milk. Food Chem..

[110] Aisyiyah Jenie S.N., Kusumastuti Y., Krismastuti F.S.H., Untoro Y.M., Dewi R.T., Udin L.Z., Artanti N. (2021). Rapid fluorescence quenching detection of *Escherichia coli* using natural silica-based nanoparticles. Sensors.

[111] Al-Awwal N., Masjedi M., El-Dweik M., Anderson S.H., Ansari J. (2022). Nanoparticle immuno-fluorescent probes as a method for detection of viable *E. coli* O157:H7. J. Microbiol. Methods.

[112] Manoharan H., Kalita P., Gupta S., Sai V.V.R. (2019). Plasmonic biosensors for bacterial endotoxin detection on biomimetic C-18 supported fiber optic probes. Biosens. Bioelectron..

[113] Song X., Wang H., Xu X. (2022). Amikacin- and AuNP-mediated colorimetric biosensor for the rapid and sensitive detection of bacteria. LWT.

[114] Verdoodt N., Basso C.R., Rossi B.F., Pedrosa V.A. (2017). Development of a rapid and sensitive immunosensor for the detection of bacteria. Food Chem..

[115] Sun F., Yan C., Jia Q., Wu W., Cao Y. (2023). A Novel Aptamer Lateral Flow Strip for the Rapid Detection of Gram-positive and Gram-negative Bacteria. J. Anal. Test..

[116] Gloag L., Mehdipour M., Chen D., Tilley R.D., Justin Gooding J., Gloag L., Mehdipour M., Chen D., Tilley R.D., Gooding J.J. (2019). Advances in the application of magnetic nanoparticles for sensing. Adv. Mater..

[117] Wang S., Zheng L., Cai G., Liu N., Liao M., Li Y., Zhang X., Lin J. (2019). A microfluidic biosensor for online and sensitive detection of *Salmonella typhimurium* using fluorescence labeling and smartphone video processing. Biosens. Bioelectron..

[118] Yang E., Li D., Yin P., Xie Q., Li Y., Lin Q., Duan Y. (2021). A novel surface-enhanced Raman scattering (SERS) strategy for ultrasensitive detection of bacteria based on three-dimensional (3D) DNA walker. Biosens. Bioelectron..

[119] Cui J., Zhou M., Li Y., Liang Z., Li Y., Yu L., Liu Y., Liang Y., Chen L., Yang C. (2021). A new optical fiber probe-based quantum dots immunofluorescence biosensors in the detection of *Staphylococcus aureus*. Front. Cell. Infect. Microbiol..

[120] Wu P., Huang R., Li G., He Y., Chen C., Xiao W., Ding P. (2017). Optimization of synthesis and modification of ZnSe/ZnS quantum dots for fluorescence detection of *Escherichia coli*. J. Nanosci. Nanotechnol..

[121] Xue L., Zheng L., Zhang H., Jin X., Lin J. (2018). An ultrasensitive fluorescent biosensor using high gradient magnetic separation and quantum dots for fast detection of foodborne pathogenic bacteria. Sens. Actuators B Chem..

[122] Hao L., Xue L., Huang F., Cai G., Qi W., Zhang M., Han Q., Wang Z., Lin J. (2020). A Microfluidic biosensor based on magnetic nanoparticle separation, quantum dots labeling and MnO_2_ nanoflower amplification for rapid and sensitive detection of *Salmonella typhimurium*. Micromachines.

[123] Gupta A., Kumari A., Kaushal N., Saifi A., Mohanta G., Sachdev A., Kumar K., Deep A., Saha A. (2022). Recent advances in the applications of carbon nanostructures on optical sensing of emerging aquatic pollutants. ChemNanoMat.

[124] Hiremath S.D., Bhosle A.A., Nayse A., Biswas S., Biswas M., Bhasikuttan A.C., Banerjee M., Chatterjee A. (2021). A redox-coupled carbon dots-MnO_2_ nanosheets based sensory platform for label-free and sensitive detection of *E. coli*. Sens. Actuators B Chem..

[125] Hassan A.H.A., Bergua J.F., Morales-Narváez E., Mekoçi A. (2019). Validity of a single antibody-based lateral flow immunoassay depending on graphene oxide for highly sensitive determination of *E. coli* O157:H7 in minced beef and river water. Food Chem..

[126] Ondera T.J., Hamme A.T. (2014). A gold nanopopcorn attached single-walled carbon nanotube hybrid for rapid detection and killing of bacteria. J. Mater. Chem. B.

[127] Mosbach K. (1994). Molecular imprinting. Trends Biochem. Sci..

[128] Piletsky S.A., Subrahmanyam S., Turner A.P.F. (2001). Application of molecularly imprinted polymers in sensors for the environment and biotechnology. Sens. Rev..

[129] Emir Diltemiz S., Denizli A., Ersöz A., Say R. (2008). Molecularly imprinted ligand-exchange recognition assay of DNA by SPR system using guanosine and guanine recognition sites of DNA. Sens. Actuators B Chem..

[130] Hayden O., Mann K.J., Krassnig S., Dickert F.L. (2006). Biomimetic ABO Blood-Group Typing. Angew. Chem. Int. Ed..

[131] Gast M., Sobek H., Mizaikoff B. (2019). Advances in imprinting strategies for selective virus recognition a review. TrAC Trends Anal. Chem..

[132] Idil N., Hedström M., Denizli A., Mattiasson B. (2017). Whole cell based microcontact imprinted capacitive biosensor for the detection of *Escherichia coli*. Biosens. Bioelectron..

[133] Asliyüce S., Bereli N., Uzun L., Onur M.A., Say R., Denizli A. (2010). Ion-imprinted supermacroporous cryogel, for in vitro removal of iron out of human plasma with beta thalassemia. Sep. Purif. Technol..

[134] Altintas Z., Guerreiro A., Piletsky S.A., Tothill I.E. (2015). NanoMIP based optical sensor for pharmaceuticals monitoring. Sens. Actuators B Chem..

[135] Gascoine P., Lacey R., Wren S.P., Sun T., Karim K., Piletsky S.A., Grattan K.T.V. (2015). Computational design and fabrication of optical fibre fluorescent chemical robes for the detection of cocaine. J. Light. Technol..

[136] Agasti S.S., Rana S., Park M.H., Kim C.K., You C.C., Rotello V.M. (2010). Nanoparticles for detection and diagnosis. Adv. Drug Deliv. Rev..

[137] Idil N., Bakhshpour M., Perçin I., Mattiasson B. (2021). Whole cell recognition of *Staphylococcus aureus* using biomimetic SPR sensors. Biosensors.

[138] Özgür E., Topçu A.A., Yılmaz E., Denizli A. (2020). Surface plasmon resonance based biomimetic sensor for urinary tract infections. Talanta.

[139] Gür S.D., Bakhshpour M., Denizli A. (2019). Selective detection of *Escherichia coli* caused UTIs with surface imprinted plasmonic nanoscale sensor. Mater. Sci. Eng. C.

[140] Çimen D., Aslıyüce S., Tanalp T.D., Denizli A. (2021). Molecularly imprinted nanofilms for endotoxin detection using an Surface Plasmon Resonance sensor. Anal. Biochem..

[141] Akgönüllü S., Armutcu C., Denizli A. (2022). Molecularly imprinted polymer film based plasmonic sensors for detection of ochratoxin A in dried fig. Polym. Bull..

[142] Akgönüllü S., Yavuz H., Denizli A. (2020). SPR nanosensor based on molecularly imprinted polymer film with gold nanoparticles for sensitive detection of aflatoxin B1. Talanta.

[143] Turkmen D., Yilmaz T., Bakhshpour M., Denizli A. (2022). An alternative approach for bacterial growth control: *Pseudomonas* spp. imprinted polymer-based surface plasmon resonance sensor. IEEE Sens. J..

[144] Bezdekova J., Zemankova K., Hutarova J., Kociova S., Smerkova K., Adam V., Vaculovicova M. (2020). Magnetic molecularly imprinted polymers used for selective isolation and detection of *Staphylococcus aureus*. Food Chem..

[145] Zhao X., Cui Y., Wang J., Wang J. (2019). Preparation of fluorescent molecularly imprinted polymers via pickering emulsion interfaces and the application for visual sensing analysis of *Listeria Monocytogenes*. Polymer.

[146] Guo Y., Li J., Song X., Xu K., Wang J., Zhao C. (2021). Label-Free Detection of *Staphylococcus aureus* based on bacteria-imprinted polymer and turn-on fluorescence probes. ACS Appl. Bio Mater..

[147] Sánchez-Cid P., Jiménez-Rosado M., Romero A., Pérez-Puyana V. (2022). Novel Trends in Hydrogel Development for Biomedical Applications: A Review. Polymer.

[148] Luo Y., Yu M., Zhang Y., Wang Y., Long L., Tan H., Li N., Xu L., Xu J. (2022). Highly sensitive strain sensor and self-powered triboelectric nanogenerator using a fully physical crosslinked double-network conductive hydrogel. Nano Energy.

[149] Su C., Huang X., Zhang L., Zhang Y., Yu Z., Chen C., Ye Y., Guo S. (2023). Robust superhydrophobic wearable piezoelectric nanogenerators for self-powered body motion sensors. Nano Energy.

[150] Luo J., Wang H., Wang J., Chen Y., Li C., Zhong K., Xiang J., Jia P. (2023). Fabrication of a High-Strength, Tough, Swelling-Resistant, Conductive Hydrogel via Ion Cross-Linking, Directional Freeze-Drying, and Rehydration. ACS Biomater. Sci. Eng..

[151] Sun X., Agate S., Salem K.S., Lucia L., Pal L. (2021). Hydrogel-Based Sensor Networks: Compositions, Properties, and Applications—A Review. ACS Appl. Bio Mater..

[152] Du X., Zhai J., Li X., Zhang Y., Li N., Xie X. (2021). Hydrogel-Based Optical Ion Sensors: Principles and Challenges for Point-of-Care Testing and Environmental Monitoring. ACS Sens..

[153] Cheng W., Wu X., Zhang Y., Wu D., Meng L., Chen Y., Tang X. (2022). Recent applications of hydrogels in food safety sensing: Role of hydrogels. Trends Food Sci. Technol..

[154] Gong J., Tanner M.G., Venkateswaran S., Stone J.M., Zhang Y., Bradley M. (2020). A hydrogel-based optical fibre fluorescent pH sensor for observing lung tumor tissue acidity. Anal. Chim. Acta.

[155] Gunda N.S.K., Chavali R., Mitra S.K. (2016). A hydrogel based rapid test method for detection of *Escherichia coli* (*E. coli*) in contaminated water samples. Analyst.

[156] Jung S., Macconaghy K.I., Guarnieri M.T., Kaar J.L., Stoykovich M.P. (2022). Quantification of Metabolic Products from Microbial Hosts in Complex Media Using Optically Diffracting Hydrogels. ACS Appl. Bio Mater..

[157] Wu P., Li G., He Y., Luo D., Li L., Guo J., Ding P., Yang F. (2020). High-efficient and sustainable biodegradation of microcystin-LR using *Sphingopyxis* sp. YF1 immobilized Fe_3_O_4_@chitosan. Colloids Surf. B Biointerfaces.

[158] Wu P., Li S., Ye X., Ning B., Bai J., Peng Y., Li L., Han T., Zhou H., Gao Z. (2020). Cu/Au/Pt trimetallic nanoparticles coated with DNA hydrogel as target-responsive and signal-amplification material for sensitive detection of microcystin-LR. Anal. Chim. Acta.

[159] Ma Y., Mao Y., Huang D., He Z., Yan J., Tian T., Shi Y., Song Y., Li X., Zhu Z. (2016). Portable visual quantitative detection of aflatoxin B1 using a target-responsive hydrogel and a distance-readout microfluidic chip. Lab Chip.

